# Automated NMR Fragment Based Screening Identified a Novel Interface Blocker to the LARG/RhoA Complex

**DOI:** 10.1371/journal.pone.0088098

**Published:** 2014-02-05

**Authors:** Jia Gao, Rongsheng Ma, Wei Wang, Na Wang, Ryan Sasaki, David Snyderman, Jihui Wu, Ke Ruan

**Affiliations:** 1 Hefei National Laboratory for Physical Sciences at the Microscale, School of Life Science, University of Science and Technology of China, Hefei, Anhui, China; 2 Pfizer Worldwide Research and Development, San Diego, California, United States of America; 3 Advanced Chemistry Development Inc., Toronto, Ontario, Canada; Beatson Institute for Cancer Research Glasgow, United Kingdom

## Abstract

The small GTPase cycles between the inactive GDP form and the activated GTP form, catalyzed by the upstream guanine exchange factors. The modulation of such process by small molecules has been proven to be a fruitful route for therapeutic intervention to prevent the over-activation of the small GTPase. The fragment based approach emerging in the past decade has demonstrated its paramount potential in the discovery of inhibitors targeting such novel and challenging protein-protein interactions. The details regarding the procedure of NMR fragment screening from scratch have been rarely disclosed comprehensively, thus restricts its wider applications. To achieve a consistent screening applicable to a number of targets, we developed a highly automated protocol to cover every aspect of NMR fragment screening as possible, including the construction of small but diverse libray, determination of the aqueous solubility by NMR, grouping compounds with mutual dispersity to a cocktail, and the automated processing and visualization of the ligand based screening spectra. We exemplified our streamlined screening in RhoA alone and the complex of the small GTPase RhoA and its upstream guanine exchange factor LARG. Two hits were confirmed from the primary screening in cocktail and secondary screening over individual hits for LARG/RhoA complex, while one of them was also identified from the screening for RhoA alone. HSQC titration of the two hits over RhoA and LARG alone, respectively, identified one compound binding to RhoA.GDP at a 0.11 mM affinity, and perturbed the residues at the switch II region of RhoA. This hit blocked the formation of the LARG/RhoA complex, validated by the native gel electrophoresis, and the titration of RhoA to ^15^N labeled LARG in the absence and presence the compound, respectively. It therefore provides us a starting point toward a more potent inhibitor to RhoA activation catalyzed by LARG.

## Introduction

Protein-protein interactions (PPIs) have recently drawn increased attention as novel therapeutic targets [1]. The small molecule inhibitors of PPIs provide us not only potential therapeutic benefits, but also finely-controlled chemical probes to the complex signal transduction pathways for a better understanding of their biological roles. Although several successful PPI inhibitors, e.g., MDM2-targeted nutlin-3 [2] and Bcl-targeted ABT-737 [3], have entered clinical trials, the discovery of PPI inhibitors remains a thorny hurdle in practice. The “hot spots” of PPIs in general are much shallow and poorly defined, thus much weaker interaction between PPI and inhibitors are expected. The application of the high throughput screening (HTS) technique in such targets is limited, as it only searches the high affinity ligands. Fragment based screening (FBS) has been emerging as an alternative approach, which starts from weakly binding hits, and then assemble those hits into highly potent inhibitors. Such intrinsically weak interactions can be readily detected by either NMR protein based chemical shift perturbation [4] or the ligand observed STD [5] and WaterLOGSY [6], [7] experiments, even at millimolar affinity levels. NMR has therefore been extensively applied in FBS to discover novel PPI inhibitors since its naissance [8].

The high hit rate of FBS can be attributed to not only the detection of weak interactions, but also the remarkably reduced chemical space for smaller compounds. For example, the number of potential lead-like compounds is estimated to be 10^9^ at the average molecular weight of 250 Da (thus dubbed fragments), but 10^20^ to 10^200^ at 500 Da [9]. Even though NMR FBS uses a remarkably reduced compound library with respect to HTS, we would still need to acquire, process and analyze thousands of spectra. Automation is clearly the key to assure consistency among screenings and logistic data management. However, the setup of a highly automated NMR FBS facility from scratch has been rarely, if at all, described in detail, thus inhibiting more extensive application of NMR FBS in a variety of research organizations.

We are particularly interested in the PPI complex of the Dbl homology (DH) domain of Leukemia Associated Rho Guanine exchange factor (LARG) and its downstream small GTPase RhoA. The LARG DH domain has the highest catalytic activity in the Dbl family to convert RhoA from the inactive GDP binding form to the active GTP binding form with 10^7^ fold enhancement [10]. The over-activation of RhoA has been found in various tumor cells [11] to regulate tumor cell adhesion [12], invasion [13] and migration [14]. LARG has also been observed to be upregulated in surgical specimens of patients with the preleukaemic disorder Shwachman-Diamond syndrome [15]. Additionally, the LARG-RhoA signal transduction pathway has also been proven to play a vital role in salt-induced hypertension [16] and the collapse of the neuronal growth cone [17]. Undesirably, the discovery of small molecule inhibitors for LARG/RhoA pathway remains difficult, since the LARG catalytic DH domain lacks a ligand binding pocket based on its crystal structure [18]. And the direct targeting of the RhoA GTP binding site has also proven challenging because of the picomolar GDP/GTP binding affinity as well as the millimolar concentration of GDP/GTP in cells [19]. This is true despite the fact that micromolar ligands have been found via virtual screening, and these compounds have later been verified and optimized [20].

A small molecule Brefeldin A has been found to trap the complex of the small GTPase Arf1 and its guanine exchange factor (GEF) to the inactivated intermediate status [21]. Recently, NMR based fragment screening has been utilized to identify small molecules which bind to GTPase RAS and inhibit the guanine exchange activity [19], [22], [23]. These discoveries inspire us to search for compounds either binding to the LARG/RhoA^.^GDP complex or RhoA^.^GDP alone using NMR fragment screening. We herein describe the step-by-step procedures of NMR FBS, controlled by our home-made automation scripts for every aspect of FBS as possible, including the construction of a fragment library, solubility determination by quantitative NMR, fragment cocktail preparation, and classified visualization of screening spectra. The application of this highly automated NMR fragment screening to DH/RhoA complex and RhoA alone identified a small molecule ligand. The chemical shift perturbation experiment demonstrates that this ligand binds to RhoA switch II region, which plays an essential role in the conversion of RhoA^.^GDP to RhoA^.^GTP. This compound blocks the formation of the DH/RhoA complex, supported by native gel electrophoresis, protein based titration of RhoA to isotope labeled DH in the absence and presence of the compound.

## Materials and Methods

### Cloning, Expression, and Protein Purification

DNA encoding the LARG DH domain (residues 766–986) was cloned and expressed with a N-terminal 6×His tag using modified PET28a (+) vectors (GE Healthcare, Shanghai, China), in which the thrombin protease sites were substituted by the TEV cleavage sites, in *E. coli* BL21(DE3)-Gold at 16°C for 20 hours. All proteins were purified on an Ni-chelating column (Qiagen, GE Healthcare) and then treated with TEV to cleave the N-terminal His tags and further purified with Ni-chelating column and size-exclusion chromotography (Superdex 200 16/60; GE healthcare). The purified DH was concentrated in the PBS buffer.

A construct encoding human RhoA (residues 1–193) was cloned into pGEX4t-1 with thrombin protease sites and expressed at 16°C in Escherichia coli BL21(DE3)-RIL. Cells were grown in either LB (Luria-Bertani) medium for unlabelled samples or minimal medium supplemented with ^15^NH_4_Cl for ^15^N-samples. The GST-fused protein was first purified using glutathione-Sepharose resins with 20 mM HEPES, 500 mM NaCl, 5 mM MgCl2, 10% glycerol, 2 mM TCEP, 0.1 mM GDP, followed by the removal of the GST tag and finally purified by gel filtration chromatography (Superdex-75 16/60, GE Healthcare). The resulting samples, used in this study, were stored in 20 mM HEPES buffer at pH 7.0 also containing 100 mM NaCl,5 mM MgCl_2_,and 5 mM TCEP.

The DH domains of LARG were then mixed with an equal molar amount of RhoA and diluted 20-fold with complex buffer (20 mM HEPES at pH 8.0, 200 mM NaCl and 2 mM TCEP). The DH/RhoA complex was finally purified by size-exclusion chromotography (Superdex 200 10/300; GE healthcare), after an incubation of 30 min at 4°C, and stored in 50 mM phosphate buffer at pH 7.0.

### Automated NMR Fragment based Screening

A commercially-available fragment library (Chembridge, San Diego, CA) was filtered according to our stringent requirements based on the compounds’ physiochemical properties, similar to the Rule of Three [24], i.e., 110≤ molecular weight ≤350, clogP≤3, number of rotable bonds ≤3, number of hydrogen bond doners ≤3, number of hydrogen bond acceptors ≤3, total polar surface area ≤110, logS_w_ (aqueous solubility) ≥ −4.5. Those properties were provided by the vendor and could also be calculated from ACD, Discovery Studio (Accelrys, Inc., San Diego, CA) or other similar software. In practice, we preferred the compounds have at least one aromatic proton peak, as the aliphatic region may suffer from the strong signals if protonated buffer was used. We eventually purchased 1008 compounds and dissolved them in DMSO-d_6_ at a concentration of 200 mM. The samples were then diluted to 1 mM for the quantitative NMR studies only in 50% D_2_O, 45% H_2_O and 5% DMSO-d_6_ (v/v). The solvent-suppressed proton spectra were acquired using our Agilent 700 MHz spectrometer equipped with a 5 mm cryoprobe and autosampler. That spectrometer allows up to 96 samples to be submitted at once and acquired in series. All spectra were stored on a network drive and processed by our custom-made automation script (ACD/Labs, Toronto, Canada). The compounds entering our final fragment library have a measured concentration higher than 0.1 mM, consistent structures, and a low percentage of impurities (<15%). The cocktail is an equal molar mixture of 10 compounds, thus at a diluted concentration of 20 mM for each individual component. In each cocktail, every pair of compounds has an acceptable degree of aromatic peak overlap, as examined by our automation script.

These cocktails were then transferred to the protein buffer, ready for NMR fragment screening. In practice, the final concentration for an individual compound may vary from around 0.2 mM to 1 mM fo different targets and for different research groups (personal communication), as it depends on the spectral sensitivity and the targeting protein’s tolerance to DMSO. In our case, the screening samples contained 0.5 mM compounds, 50% D_2_O, 45% H_2_O and 5% DMSO-d_6_, and were prepared either in the absence of target proteins as a negative control of NMR fragment screening, or in the presence of 10 µM RhoA alone or DH/RhoA complex in the aforementioned complex buffer for primary fragment screening. As some compounds have an aqueous solubility less than 0.5 mM, the screening samples may contain precipitations, which can be filtered out or centrifuged to the bottom of the sample tubes. Three 1D spectra, i.e., ^1^H WATERGATE (w5) [25], Saturation Transfer Difference (STD) [26], and WaterLOGSY [6], were acquired for each sample. The acquisition parameters were optimized using the model sample containing 0.1 mM BSA, 1 mM sucrose and 1 mM tryptophan. The WATERGATE spectrum was acquired for 2.4 minutes with the acquisition and relaxation times both set to 2s and 32 scans. The STD experiment was acquired using the acquisition time of 1 s, 32 dummy scans, and relaxation delay of 0.1 s, followed by a 2 s Gauss pulse train with the irradiation frequency at −0.7 ppm or −50 ppm alternatively. The total acquisition time was 15 minutes with 256 scans. WaterLOGSY was acquired for 15.1 minutes with 1 s acquisition time, 1 s relaxation and 1.3 s NOE mixing time and 256 scans.

The spectra were processed in real time by the automation script and updated to our database, where each database entry, called a record, contains the three screening spectra for that cocktail, as well as the reference proton spectra for each individual component previously acquired for concentration measurement. Such visualization for each record allows a straightforward identification of possible hits. The hits were singled out for positive results in both STD and WaterLOGSY. A secondary screen followed using the same NMR experiment for each compound prepared individually.

### NMR HSQC Titration

The 0.5 mM ^15^N-labelled RhoA sample was titrated by the small molecule at the molar ratio of compound/RhoA of 0.1, 0.25, 0.5, 1.0, 2.0, 5.0, respectively. The HSQC spectrum was collected for each titration point in our Agilent 500 MHz spectrometer equipped with RT probe, with a total experimental time of 38 minutes, relaxation delay of 1 s and 64 increments in ^15^N dimension and 16 scans per increment.

The 500 µL 0.2 mM ^15^N DH sample was first incubated with 5 µL compound (200 mM stock solution in DMSO-d6, final concentration of 2 mM) or equal volume DMSO-d6, respectively. These two samples were then titrated by the unlabeled RhoA at a molar ratio of RhoA/DH of 0.0, 0.1, 0.2, 0.4, 0.8, 1.0, respectively. At each point, a TROSY-HSQC spectrum [27] was collected at a total experimental time of 38 minutes, with the relaxation delay of 1 s, 64 increments in ^15^N dimension and 16 scans per increment in the Agilent 700 MHz spectrometer.

## Results and Discussion

Our intention in general is to build a quick and streamlined NMR binding assay and hit validation, which would allow us to evaluate the druggability of a variety of potential targets. Meanwhile, the identified hits can act as a good starting point for the future screening over a subset of the HTS library containing the substructures analogous to the identified hits. Towards this goal, we automated every procedure of NMR fragment screening as possible from the initial library construction till the identification of a small molecule to targeting the DH/RhoA^.^GDP complex, which are separated into the following modules.

### 1 Small but Versatile Fragment Library

**Figure 1:**
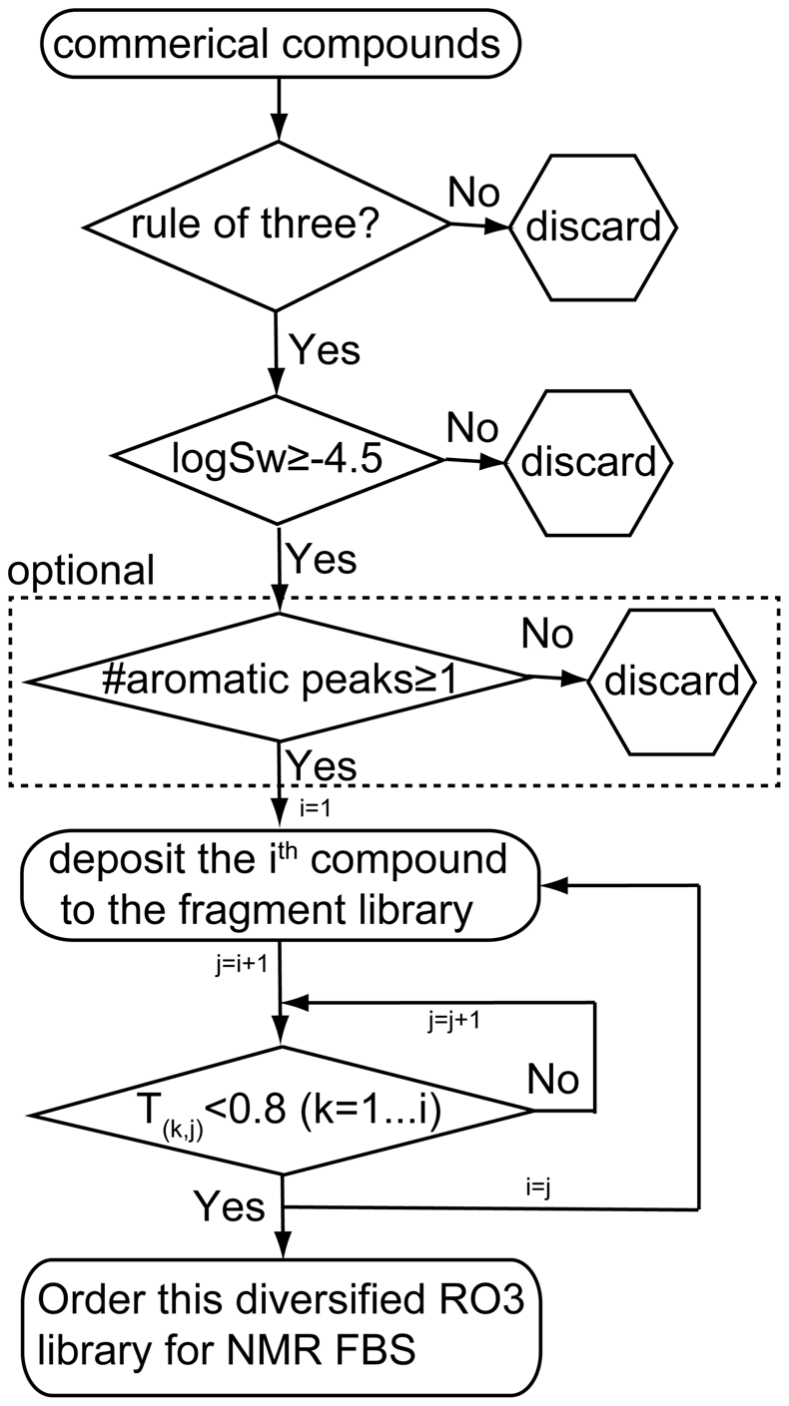
The scheme to build a small but diverse fragment library from commercial available compounds.

The commercially available compounds were first filtered by the well-known Rule of Three principle [24], including the extended empirical rule of the predicted aqueous solubility logS_w_ ≥ −4.5 as well as the optional requirement of at least one aromatic proton, as illustrated in Figure 1 and Script S1 in File S1. Diversity among the compounds is highly desirable to cover a wide chemical space, we therefore ensured that any pair of compounds in our library has a Tanimoto similarity score lower than a user defined threshold. As shown in Figure 1, for any new compound to be added to the existing library, its pairwise Tanimoto score with the present library members was calculated by the Open Babel program to compare their molecular fingerprint, externally called in our script S2 in File S1. If any of these scores is above the threshold, 80% in our case, such compound will be excluded from the fragment library. Following such procedure, we eventually generated a fragment library of 1008 compounds, which fulfilled all of the above requirements.

### 2 Automated Determination of the Aqueous Solubility

The experimentally determined aqueous solubility is a prerequisite to avoid the misinterpretation of screening and does-dependent assay results. As is well known, the sample concentration is proportional to the signal intensity per proton when using the same NMR acquisition parameters. The key remaining issue is thus to determine the total number of protons (defined as H_T_) within the area of interest (AOI) [28]. Although the integrals can be defined by NMR experts to match the proposed structure, such a laborious procedure can be well undertaken by computers, especially for a large amount of NMR data to be processed. More recently, the automated concentration determination has been successful for 80% of pharmaceutical compounds even in the absence of structures [28], it may, however, fail to estimate the integrals for symmetrical compounds or some fragments, which may only have couple of peaks within AOI.

**Figure 2:**
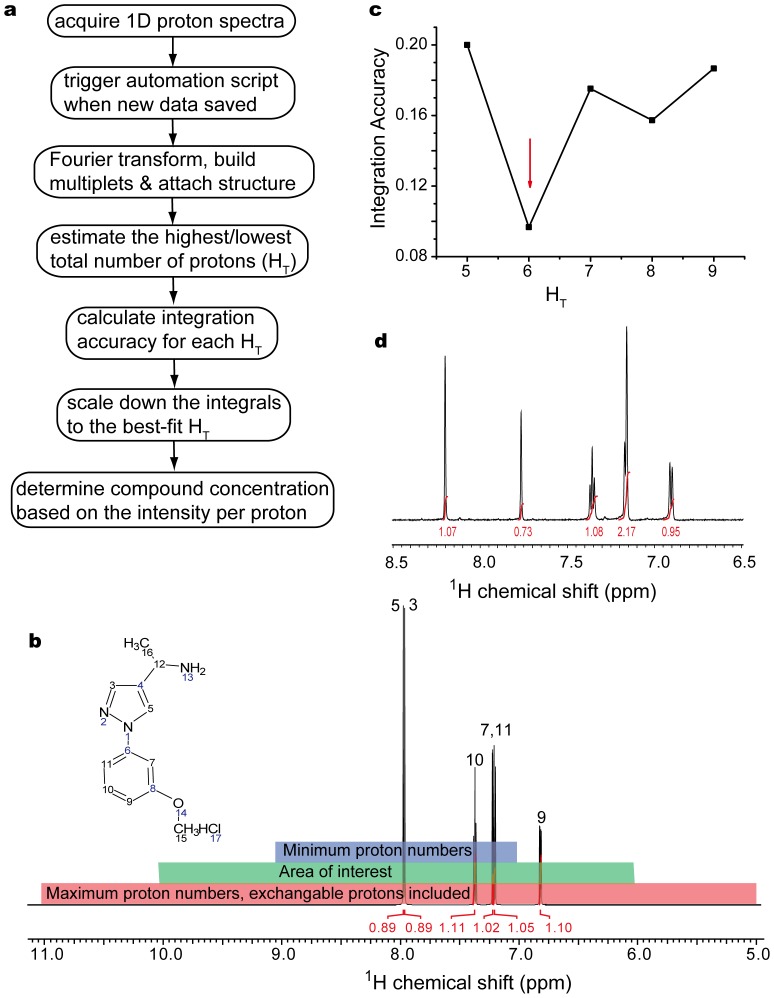
Determination of the aqueous concentration of fragments by automated quantitative NMR. a) The flow path of the processing and automated concentration measurement from 1D proton spectra. b) The predicted proton spectrum of a typical fragment compound with the area of interest in green, where the minimum (maximum) of H_T_ was the number of protons counted in the purple and red area, respectively. The exchangeable protons are included only in the estimation of maximum proton numbers. c) The integration accuracy for an experimental spectrum correlates with the total number of protons (H_T_) within the area of interest, where the arrow indicated the best-fit value for H_T_. d) The experimental spectrum with the total integrals within AOI given by the best-fit H_T_.

We hence first developed an algorithm to estimate the upper and lower limit of H_T_ from the predicted spectrum. As demonstrated in Figure 2a and Script S3 in File S1, the 1D WATERGATE spectra were acquired for each compound at the nominal 1mM concentration, in the batch mode handled by the 96 well autosampler. Once the data were deposited to the network drive, our automation script would be triggered to process the data, including the conventional processing prior to and post Fourier transformation and multiplicities analysis [29], as well as the attachment of the compound’s structure retrieved from the database of the fragment library. The proton spectrum was predicted using the ^1^H structure validation module of ACD from the proposed structure [29]. As exemplified in Figure 2b, we deliberately set AOI to 6∼10 ppm to avoid the signal interference in the aliphatic region from DMSO and/or protonated buffers if used, e.g., TRIS or HEPES. The minimum of H_T_ was set to the number of non-exchangeable protons in the predicted spectrum within the range of 7 to 9 ppm (purple area in Figure 2b), to accommodate the prediction deviation up to 1 ppm (also a user adjustable parameter), taken into account a standard deviation of ca. 0.3 ppm for ^1^H chemical shift prediction over 116,000 compounds [30]. The maximum of H_T_ was determined in a similar way but within the range of 5 to 11 ppm (red area in Fig. 2b), including all exchangeable protons whose chemical shifts were difficult to predict.

With the range of H_T_ determined, we then went over every possible H_T_ and determined its best-fit value such that the integrals are as close to their nearest integers as possible in the experimental spectrum. As illustrated in Figure 2c, the experimental spectrum was reintegrated for every enumerated H_T_. At the best-fit H_T_, the integrals on average should be as close to their nearest integers as possible. We defined such cost function as integration accuracy, i.e., the root mean squared deviation of the integral (a real number) of the peaks from their nearest integers correspondingly (Script S3 in File S1). The H_T_ at the minimum of integration accuracy would allow the best estimation of the proton numbers, as exemplified in Figure 2d. The intensity per proton once compared with that of a pure and soluble compound with known concentration, would allow the measurement of the fragment concentration, or its aqueous solubility if precipitated.

This approach has been previously applied to determine the concentration of prototypical compounds in the pharmaceutical company with an accuracy of ca. 15% (data not shown). We choose the threshold of 0.1 mM (a user defined parameter in Script S4 in File S1) to exclude the compounds with lower solubility, in order to allow a sufficient NMR sensitivity for concentration measurement, and a sufficient solubility for the following affinity assay as well. Such cutoff coincides with the extended Lipinski Rule of Five [31], which is reasonable as these initial fragments are expected to have a higher solubility than the later developed drug-like compounds. The final spectra, scaled down to the best-fit H_T_ as illustrated in Figure 2d, were interrogated by human experts to check impurity level (<15%) and structure consistency. In a word, the predicted integrals agree well with the ones estimated by human experts, and we observed a clear correlation between the precipitated samples and the aqueous concentrations lower than 0.5 mM. Eventually, a total of 893 compounds from the purchased 1008 compounds passed the solubility criterion and human inspection, and were uploaded to our fragment library (Script S4 in File S1) for following screenings.

### 3 Dispersed Fragment Cocktails

**Figure 3:**
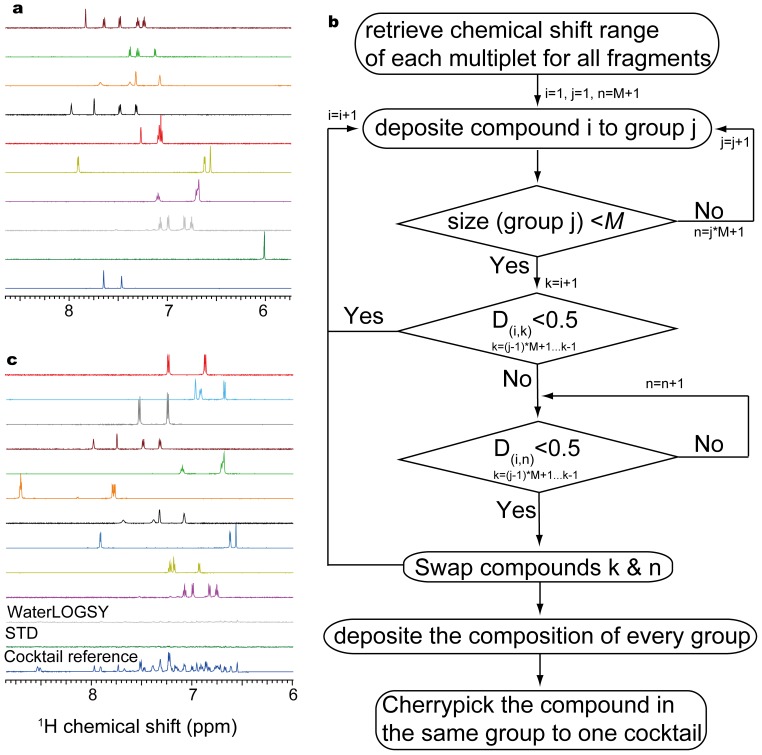
The preparation of dispersed fragment cocktails. a) The spectra of 10 fragments in an aliphatic order of the compound IDs. b) The scheme of grouping compounds with mutual dispersed spectra. c) The typical components of a cocktail (top ten spectra) prepared based on the grouping strategy. The cocktail 1D proton, STD and WaterLOGSY spectra (three bottom ones) were acquired for every cocktail in the absence of proteins as a negative control.

The throughput of NMR FBS can be significantly enhanced using compound cocktails, the detailed procedure of the cocktail preparation, however, remains elusive in literatures. A random mixing of the compounds, as exemplified in Figure 3a, may render severe peak overlapping, which would make the identification of the right hits difficult.

We hence developed a protocol for the cocktail preparation with dispersed proton spectra. Figure 3b and Script S5 in File S1 describe our algorithm to group the 893 compounds at M compounds per cocktail, with a pairwise peak overlapping below a certain threshold for every two compounds within the same cocktail. In our setting, 10 compounds per cocktail would allow a well dispersed spectrum, consistent with the normally used 8 to 15 compounds [32]. Considering that the fragment screening hit rates are normally less than 10% and even much lower for PPI targets [33], the probability of having three hits in the same NMR cocktail is quite slim. It is, therefore, sufficient if we can discern pairwise compounds in the same cocktail. We hence introduce the pairwise dispersity *D_(i,k)_* for compound *i* and *k* within AOI as
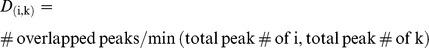
where the peak overlapping is counted for the chemical shift range of each multiplet of compound *i* partially overlapped with that of compounds *k*. The multiplets were constructed using the same ACD module as previously illustrated in Figure 2d with the chemical shift range defined across the peak. Thus, *D_(i,k)_* at 0.5 means at least 50% of peaks are distinct for either compound *i* or *k*, which should allow the unambiguous identification of fragment hits. We hence separated the 893 compounds in 90 groups, other than the first compound in the goup *j*, the next compound *k* was compared with the existing members within the same group. If the dispersity scores were all below the threshold of 0.5, this compound remained in the group. Otherwise, we searched the compounds in the following groups till one compound can meet such criterion, which would then be swapped with compound *k*. The above procedure was repeated for the rest compounds and groups. We then picked the compounds in the same group to one cocktail in the standard 96 well rack. During the preparation of the present article, a computer-aided design of the NMR fragment mixtures has been dedicated to the minimization of the global peak overlap using a Monte Carlo approach [34]. Figure 3c demonstrates that our approach was also sufficient to achieve a well dispersed cocktail spectrum.

### 4 Fragment Screening over DH/RhoA Complex

As a negative control of the fragment screening, the cocktails were first screened in the absence of target proteins using 1D proton, ligand based Saturation Transfer Difference (STD) and WaterLOGSY spectra. Theoretically, STD spectra show no signals while WaterLOGSY spectra give signals at the same sign except for the exchangeable protons. Such a negative control allows a calibration of NMR parameters to avoid the undesired excitation of small molecular signals in STD, and the identification of exchangeable proton signals in WaterLOGSY for the whole library. The processing of the screening spectra was automated in a way as shown in the first three steps of Fig. 2a. For a better visualization of the screening results as shown in Figure 3c, we also deposited the three cocktail spectra along with the ten reference spectra for corresponding components into our database (see also Script S6 in File S1). Three out of the 90 cocktails (3.3%) demonstrated lots of absorptive peaks above the noise level in the aromatic regions of STD spectra. It can be ascribed to compound aggregation (precipitation observed in these three cocktails), and other researchers also observed that less than 5% of their fragments may form micelle at high concentration as validated by dynamic light scattering (personal communication). The other reasons are the cancelation artifacts and the undesired saturation for the fragments with chemical shifts near the irradiation frequency, although it is less likely since our irradiation frequency at −0.7 ppm was at least 1 ppm away from those methyl peaks. Such undesired saturation could also be alleviated if we rely more on the aromatic peaks in STD to identify hits.

**Figure 4:**
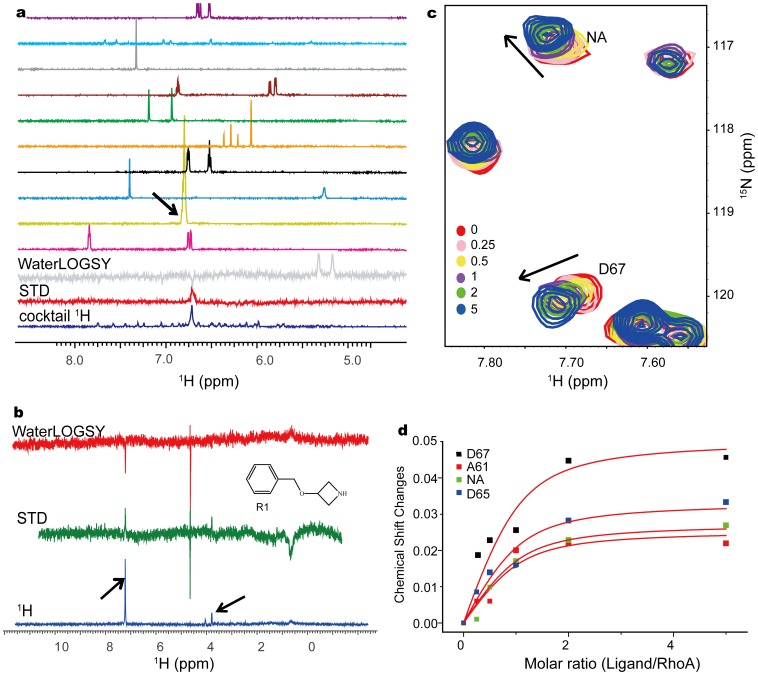
Fragment screening over DH/RhoA complex. a) The primary screening result for one typical cocktail, with 10 reference spectra and 3 screening spectra depicted. The arrow indicates the identified hit; b) The secondary screening over the identified hit R1 individually. c) The chemical shift perturbation in ^15^N RhoA HSQC spectra induced by titration of the compound R1 with the molar ratio varying from 0.0 to 5.0, as the numbers denoted. The perturbed residues D67 and NA (not assigned) are labeled with the arrows pointing out the trend of chemical shift changes. d) The affinity of R1 to RhoA can be best-fitted from the dose-dependent titration curves for the four disturbed residues simultaneously. Chemical shift changes were calculated as [(Δδ_H_)^2^+(Δδ_N_/5)^2^)]^1/2^, where Δδ_H_ and Δδ_N_ denote the chemical shift changes for ^1^H and ^15^N, respectively.

The first attempt of fragment screening over 1∶1 DH/RhoA complex was thereafter applied using the same spectroscopic setting as that of the negative control. Since our fragment screening is designed for multiple targets, which may prefer different pH and buffers, e.g., PBS, Tris or HEPES, the ^1^H spectrum acquired in H_2_O/D_2_O for concentration measurement was directly used here as a reference. The chemical shift variation in the reference and screening spectra due to different buffer conditions used, as well as the low sensitivity of STD and WaterLOGSY spectra, makes the full automation for the identification of fragment hits very challenging in practice. We, therefore, only automated the data processing and displayed the screening spectra along with the corresponding reference one (see Script S6 in File S1), in an intuitive way as illustrated in Figure 4a. The signals above the noise level in STD were first picked out and confirmed by peaks at the same chemical shifts in WaterLOGSY. As the signal intensities could vary significantly even for the same hit in STD [26] and/or WaterLOGSY [6], we rely on the whole pattern of chemical shift distribution and multiplicities determined in the cocktail ^1^H spectrum to identify the most possible hits. For instance, compound R1 gives one degenerated broad signal at 7.30 ppm in the aromatic region in H_2_O/D_2_O as shown in Figure 4a (structure shown in Figure 4b, see also Figure S1 for its full proton spectrum in DMSO-d6), which matches the degenerated peak at 7.22 ppm in STD and WaterLOGSY spectra. Other peaks in WaterLOGSY arising from the direct interaction between H2O and ligand cannot be confirmed by STD and thus ignored. Benefitting from the dispersed cocktail spectra, we found 7 hits from this first round of fragment screening without the need of further deconvolution over each component (See Figure S2a–f for the spectra of rest six compounds picked out by this primary screening).

These 7 hits were thereafter confirmed by the secondary screening for each individual compound. As illustrated in Figure 4b and S2g-l, the primary cocktail screening can be compared with the corresponding secondary screening for the individual hit, and visualized in the same database entry (see Script S6 in File S1). Firstly, we can easily verify whether the right hit is identified as the same chemical shifts were observed for the two screenings. The deconvolution of those seven hits were all confirmed, validated our strategy of the cocktail preparation with mutual dispersed spectra. Secondly, the secondary screening for the individual compound would exclude the false positive hits arising from compound aggregation in the cocktail, and/or cancelation artifacts. Except for the two compounds shown in Figure 4b and Figure S2g, the rest five compounds did not present signals in both STD and WaterLOGSY spectra in the secondary screening (see Figure S2h-l) and thus excluded from further affinity assay.

We have also applied the automated NMR fragment screening over RhoA^.^GDP alone. Eleven compounds were picked out from the primary screening (top three spectra in Fig. S3a–j), where five of them (Figure S3a–d), including compound R1 (intrinsically the same spectra as shown in Figure 4a–b, thus not shown repeatedly), were confirmed in the secondary screening over individual hit. Our observation for RhoA alone and DH/RhoA complex both demonstrated the necessity to run a secondary screening to identify the most possible hits from the primary one.

### 5 Hit Validated as the Blocker of the Complex Formation

The binding site and affinity of compound R1 from the secondary screening of RhoA/DH complex and RhoA alone was then determined by the dose dependent chemical shift perturbation for DH and RhoA alone, respectively, a widely applied approach for FBS hit validation [4]. We did not observe a submillimolar affinity of R1 to DH alone, which is also consistent with our fragment screening over DH alone (no submillimolar hits identified possibly due to the shallow surface of DH, data not shown). Figure S4 depicts the superimposed full spectra of RhoA (Figure 4a) and the zoomed area (Figure 4c and Figure S4b) upon titration of R1 at the molar ratio from 0.0 to 5.0. We did not further increase the amount of R1 as we observed a slight variation of chemical shift changes from the molar ratio of 2.0 to 5.0. And higher concentration of R1 may also introduce more DMSO (compounds dissolved DMSO-d6) to destabilized RhoA. The titration of compound R1 to the 1∶1 DH/RhoA complex remains experimentally difficult because of the poor complex HSQC spectra (Figure S5f) in comparison with that of RhoA (Figure S4a) or DH alone (Figure S5a), which could be attributed to the larger molecular weight (LARG DH-PH/RhoA complex crystallized as a tetramer of heterodimer, associated by the DH-DH interface [18], PDB: 1X86) and possible exchange events. The hit R1 disturbs the residues near the switch II region of RhoA, i.e., A61, D65, D67 and an unassigned residue missing in the NMR assignment (residues 1–181) [35], probably the extended C-terminal residues in our full-length RhoA. The binding affinity of 0.11 mM was determined from the dose dependent chemical shift changes of the four residues (Figure 4d).

**Figure 5:**
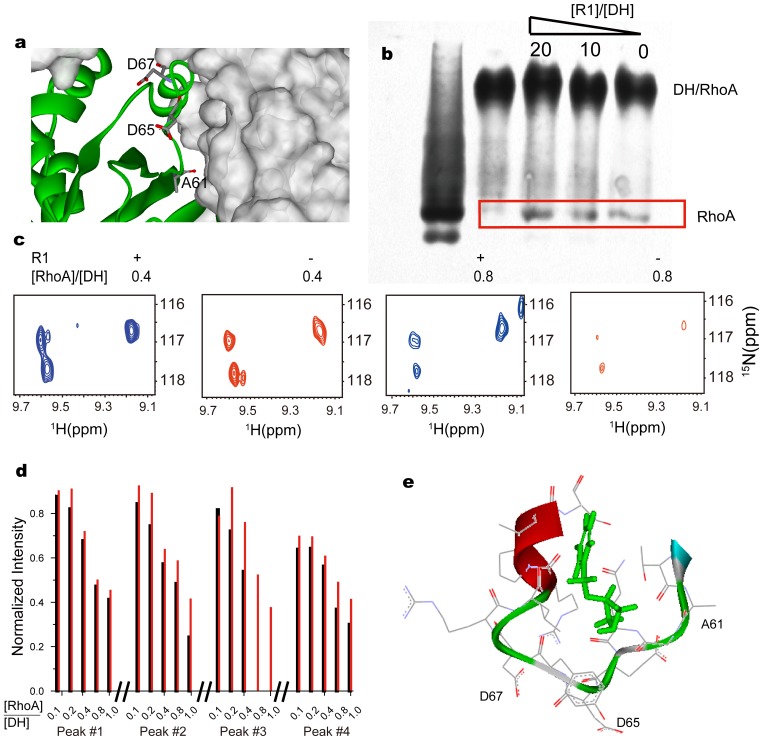
Compound R1 blocks the formation of DH/RhoA complex. a) Perturbed residues (stick representation and labeled) mapped to the DH (grey surface) and RhoA (ribbon) complex. b) The native gel electrophoresis running at pH 8.3 of RhoA alone (first lane) and DH/RhoA complex at the concentrations of 37.5 and 30 µM, respectively (second to fifth lane). The compound R1 was added at the denoted molar ratio to DH for the rightmost three lanes with equal amount of DMSO added, where red box highlighted the band for RhoA monomer. c) The 0.2 mM ^15^N DH were first incubated with either 2 mM compound R1 or DMSO, and then subjected to the titration of non-labeled RhoA. Typical trosy HSQC spectra for these two samples (+/− denotes the presence/absence of R1, respectively) were depicted at the RhoA/DH molar ratio of 0.4∶1 and 0.8∶1 as denoted, using the same display setting. For clarity, the spectra in the presence/absence of R1 were plotted in blue and red, respectively. d) Correlation between peak intensities of ^15^N DH and RhoA/DH molar ratio, in the presence of DMSO (black lines) and compound R1 (red lines), respectively. Peaks 1–4 (numbered in Figure S5g) were separated by double lines. The intensities were normalized over those corresponding peaks in the absence of RhoA for each sample. Peaks with intensity less than 3 fold noise level were ignored. e) Simulated RhoA/R1 interactions using the CDOCKER scheme, where the binding site was defined as a sphere to accommodate all perturbed residues.

The perturbed residues were plotted on the ribbon representation of RhoA using the DH/RhoA complex (PDB: 1X86), as depicted in Figure 5a. Clearly, R1 binds to the switch II region of RhoA right on the DH/RhoA complex interface, which plays a critical role for the conversion of RhoA^.^GDP to the activated RhoA^.^GTP catalyzed by its GEF. It then brings us an interesting question of whether R1 would function like Brefeldin A to trap the DH/RhoA to the inactivated complex form, or it would pry DH from the RhoA surface and thus block the complex formation. Both could in principle modulate the GEF activity of DH to RhoA.

We therefore detected the amount of free RhoA in the 0.8∶1.0 DH/RhoA solution by the native 12% gel electrophoresis running at pH 8.3, where free RhoA (estimated pI of 5.83) and DH/RhoA complex are negatively charged while DH (estimated pI of 9.24) is positively charged and thus invisible. This experiment was repeated three times and the result consistently demonstrates that the amount of free RhoA is increased upon the addition of compound R1 (Figure 5b), indicating that R1 may actually inhibit the formation of the DH/RhoA complex.

A more quantitative assay for the complex formation is to monitor the HSQC signal intensities of ^15^N DH, upon the titration of the unlabeled RhoA for two separate DH samples, either incubated *a priori* with 10 fold excess of R1 or not. The full trosy HSQC spectra of DH at various RhoA/DH molar ratios were illustrated in Figure S5a–f in the presence of DMSO, and Figure S5g-l in the presence of compound R1. Figure 5c depicted the spectra of typical peaks enlarged for clarity at the RhoA/DH molar ratio of 0.4 and 0.8 and for these two samples, respectively. Taken into account the low sensitivity for the 1∶1 DH/RhoA complex (Figure S5f), we may roughly assume that signal intensities were mostly arising from free DH, i.e., the concentration of free DH is roughly proportional to the signal intensities [36]. That is to say, the signal intensities were expected to be higher in the presence of R1, assuming R1 could bind RhoA to block the formation of DH/RhoA complex.

The intensities of thirteen isolated peaks (randomly picked out and numbered in Figure S5g) were, thereafter, plotted against the RhoA/DH molar ratio, in the presence and absence of R1, as illustrated in Figure 5d and Figure S6. For a better comparison, the intensities for each sample at various RhoA/DH molar ratios were normalized over the initial ones prior to the titration of RhoA, i.e., the spectra in Figure S5a and Figure S5g accordingly. Clearly, the signal intensities decreased remarkably upon the titration of RhoA in the presence of R1 indicating the formation of DH/RhoA complex, and residues at the complex interface could possibly relax even faster. While in the absence of R1, the intensities are in general higher, indicating a higher portion of DH monomer in solution. As a rough estimation, the binding affinity between DH and RhoA was estimated as 5 µM from the intensity changes upon titration of RhoA in the absence of compound R1 [36], neglecting the effect of chemical exchanges. It is hence reasonable to observe that ten fold excess of R1, albeit with 110 µM affinity to RhoA, could compete effectively with DH to block the DH/RhoA complex formation.

The role of compound R1 was also supported by docking compound R1 to RhoA surface using the CHARMm-based molecular dynamics scheme [37], where the proposed protein-ligand interactions were depicted in Figure 5e. The simulation results indicate that compound R1 may compete with DH to bind the site in Switch II of RhoA. It is also consistent with the recent discovery of small molecules inhibiting the guanine exchange activitiy of RAS by NMR FBS [19], [22], [23]. Meanwhile, Shang et. al. have recently identified a 0.4 µM affinity inhibitor by virtual screening, which binds to the surface sandwiching Trp 58 of RhoA, via two aromatic rings tethered by a linker [38]. It inhibits the RhoA activation catalyzed by its GEF and RhoA-regulated functions in cell. Our compound R1 binds to only part of the surface areas, thus a lower affinity as expected. Its structure, however, is remarkably different from any of their lead compounds, underpining the power of NMR fragment based screening to sample wider chemical spaces. This compound hence opens a new route towards the potent inhibitors targeting the DH/RhoA protein-protein interaction.

## Conclusions

We have described the details of an automated NMR fragment based screening protocol, from the construction of the fragment library, aqueous solubility measurement, and cocktail optimization till the screening of DH/RhoA complex and RhoA alone. Two fragment hits were identified from the primary and secondary screening of the DH/RhoA complex. Using HSQC titration experiments, we found that one hit at 0.11 mM affinity binds to the cavity adjacent to the switch II region of RhoA. This compound inhibits the formation of DH/RhoA complex, supported by native gel electrophoresis and more quantitatively, the titration of unlabeled RhoA to ^15^N DH in the presence and absence of this small molecule, respectively. We are now working on the evolution of the hit into a more potent inhibitor of the DH/RhoA complex and evaluation of its role to regulate LARG/RhoA pathway.

### Supporting Information Available

Included in the supporting information is the home-made automation scripts (Script S1–S6 in File S1) and more NMR spectra of the compounds, RhoA alone and DH/RhoA complex (Figure S1–S6).

## Supporting Information

Figure S1 The proton spectrum of compound R1 in DMSO-d6.
(TIF)

Figure S2 **The primary screening spectra for DH/RhoA complex (aromatic region only for clarity, top ten reference spectra and buttom three screening spectra in a–f, respectively) and the secondary ones for the six hits correspondingly (full spectra in g–l, respectively).**
(TIF)

Figure S3 **The primary (top three) and secondary screening (buttom three) spectra for RhoA alone.**
(TIF)

Figure S4 **The superimposed full (a) and zoomed (b) 1H-15N HSQC spectra of upon the titration of compound R1 at the molar ratio ([R1]/[RhoA]) from 0.0 to 5.0, as the numbers denoted.** The perturbed residues are labeled with arrows indicating the trend of chemical shift changes. Zoomed area in the black box (dotted line) is displayed in Figure 4c.
(TIF)

Figure S5 **1H-15N HSQC spectra of 15N DH upon the titration of unlabeled RhoA at the molar ratio ([RhoA]/[DH]) of 0.0. 0.1, 0.2, 0.4, 0.8, 1.0 in the absence of R1 (using DMSO as a control, a to f respectively) and in the presence of R1 (g to l accordingly).**
(TIF)

Figure S6 **Correlation between peak intensities of 15N DH and RhoA/DH molar ratio, in the presence of DMSO (black lines) and compound R1 (red lines), respectively.** Peaks 5–13 (numbered in Figure S5g) were separated by double lines. The intensities were normalized over those corresponding peaks in the absence of RhoA for each sample. Peaks with intensity less than 3 fold noise level were not measured.
(TIF)

File S1 **This file includes Scripts S1–S6.** Script S1.ACD/Automation script for filtration of commercial available fragment compounds based on the Rule of Three. **Script S2.** Automation script for the exclusion of compounds with a high Tanimoto similarity score in comparison with any existing member of the fragment library. **Script S3.** Script for thedetermination of the aqueous solubility concentration. **Script S4.** Upload the compounds with appropriate aqueous solubility and impurity levels to the final screening database. **Script S5.** Preparation of fragment cocktails with dispersed proton spectra. **Script S6.** Script for the processing and visualization of the fragment spectra of Watergate, STD and WaterLOGSY.
(DOC)
