# Supplementary figures and images for: Automated NMR Fragment Based Screening Identified a Novel Interface Blocker to the LARG/RhoA Complex
Source: PLoS One. 2014 Feb 5;9(2):e88098. doi: 10.1371/journal.pone.0088098 (PMC3914932; doi:10.1371/journal.pone.0088098)

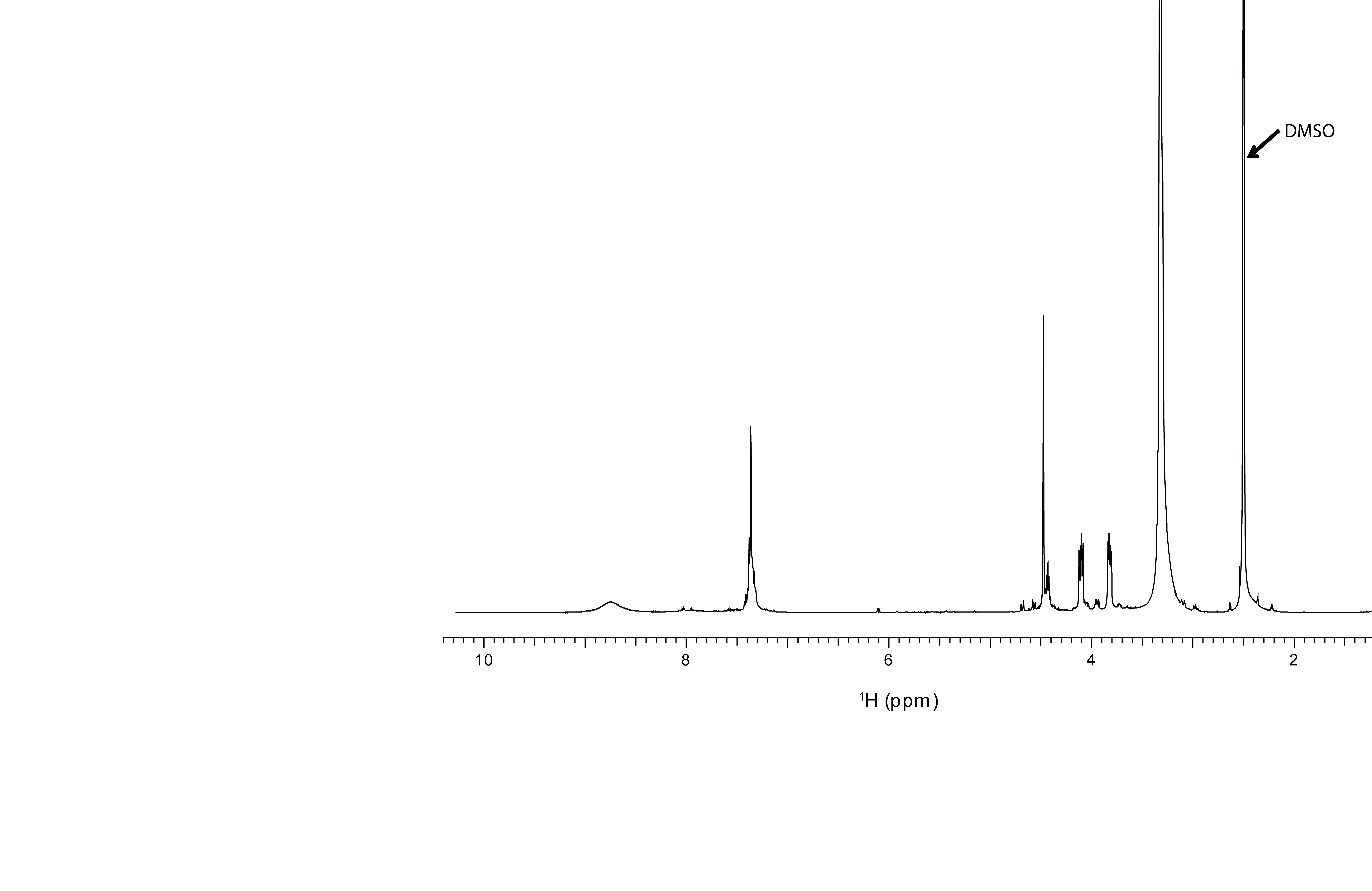

Supplement: Figure S1 — The proton spectrum of compound R1 in DMSO-d6. (TIF) [file pone.0088098.s001.tif]

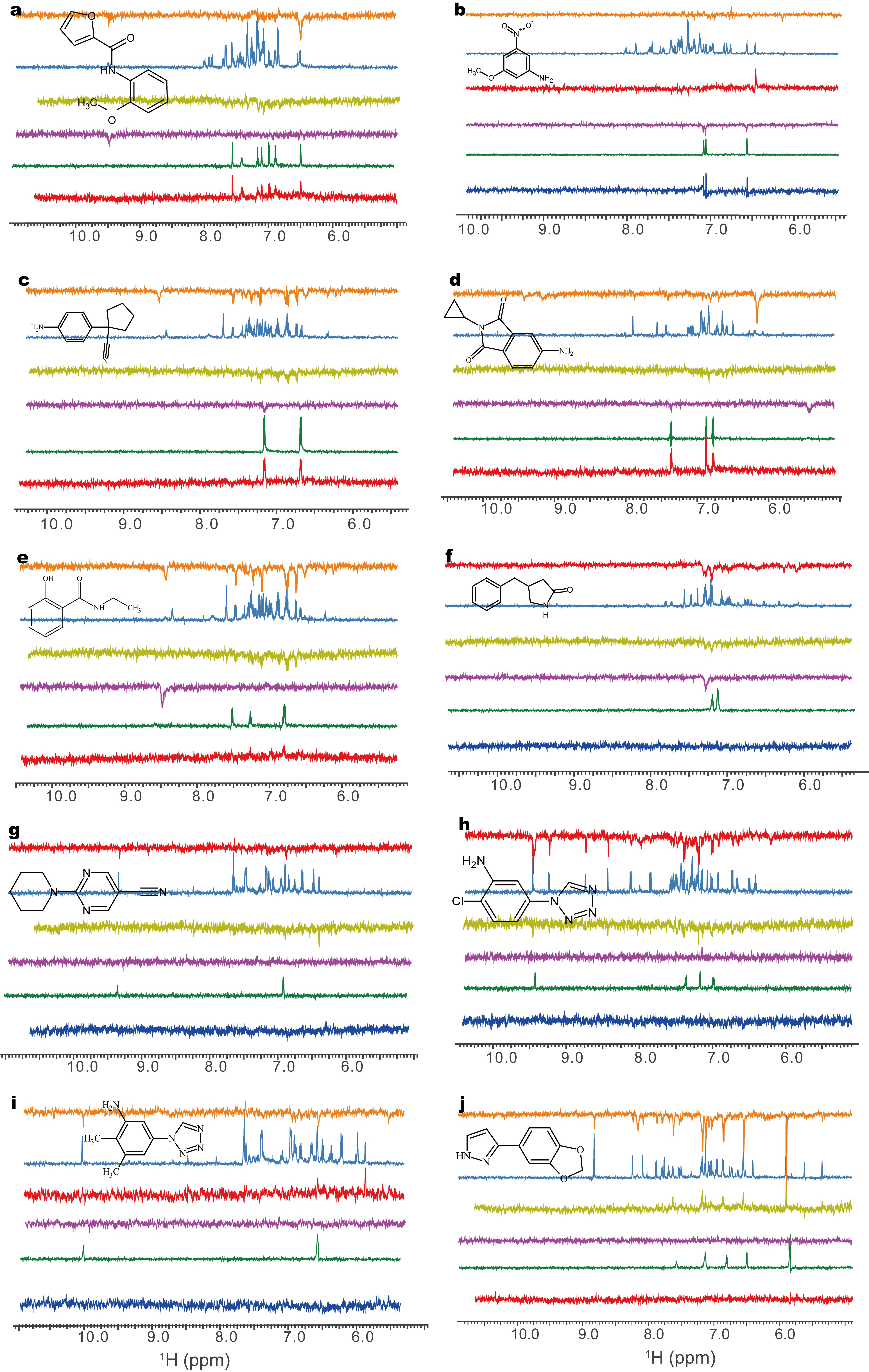

Supplement: Figure S3 — The primary (top three) and secondary screening (buttom three) spectra for RhoA alone. (TIF) [file pone.0088098.s003.tif]

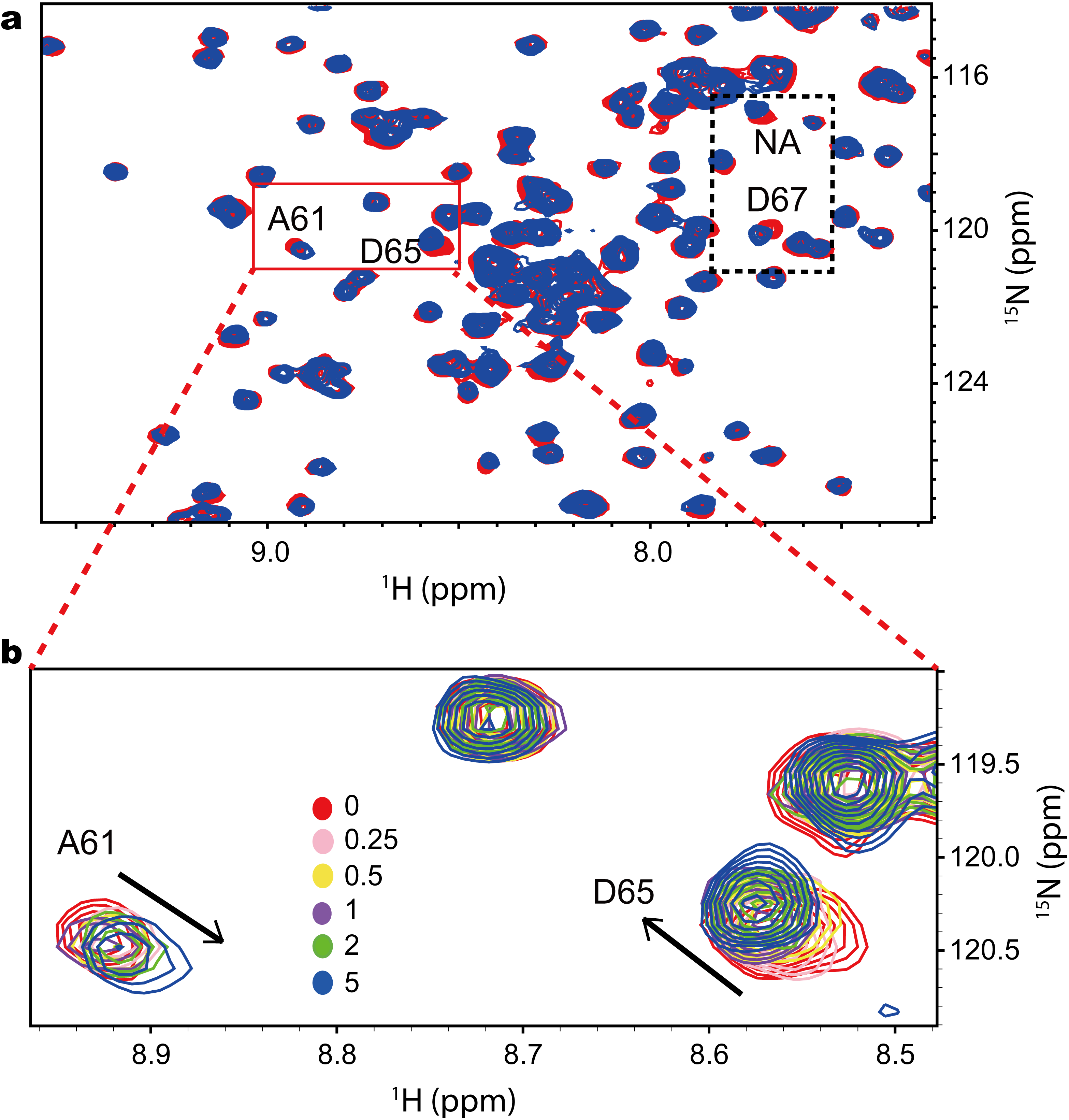

Supplement: Figure S4 — The superimposed full (a) and zoomed (b) 1H-15N HSQC spectra of upon the titration of compound R1 at the molar ratio ([R1]/[RhoA]) from 0.0 to 5.0, as the numbers denoted. The perturbed residues are labeled with arrows indicating the trend of chemical shift changes. Zoomed area in the black box (dotted line) is displayed in Figure 4c. (TIF) [file pone.0088098.s004.tif]

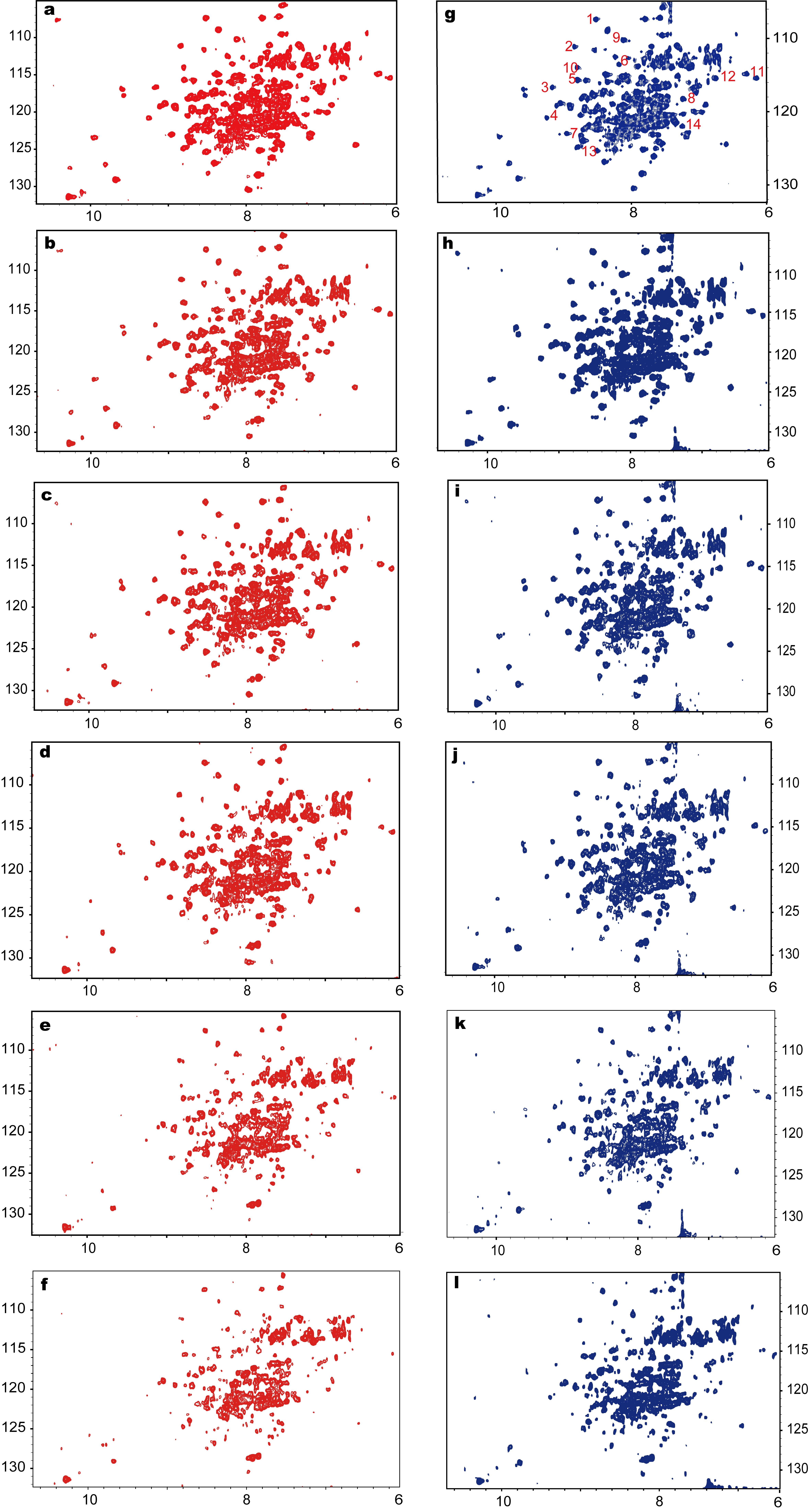

Supplement: Figure S5 — 1H-15N HSQC spectra of 15N DH upon the titration of unlabeled RhoA at the molar ratio ([RhoA]/[DH]) of 0.0. 0.1, 0.2, 0.4, 0.8, 1.0 in the absence of R1 (using DMSO as a control, a to f respectively) and in the presence of R1 (g to l accordingly). (TIF) [file pone.0088098.s005.tif]

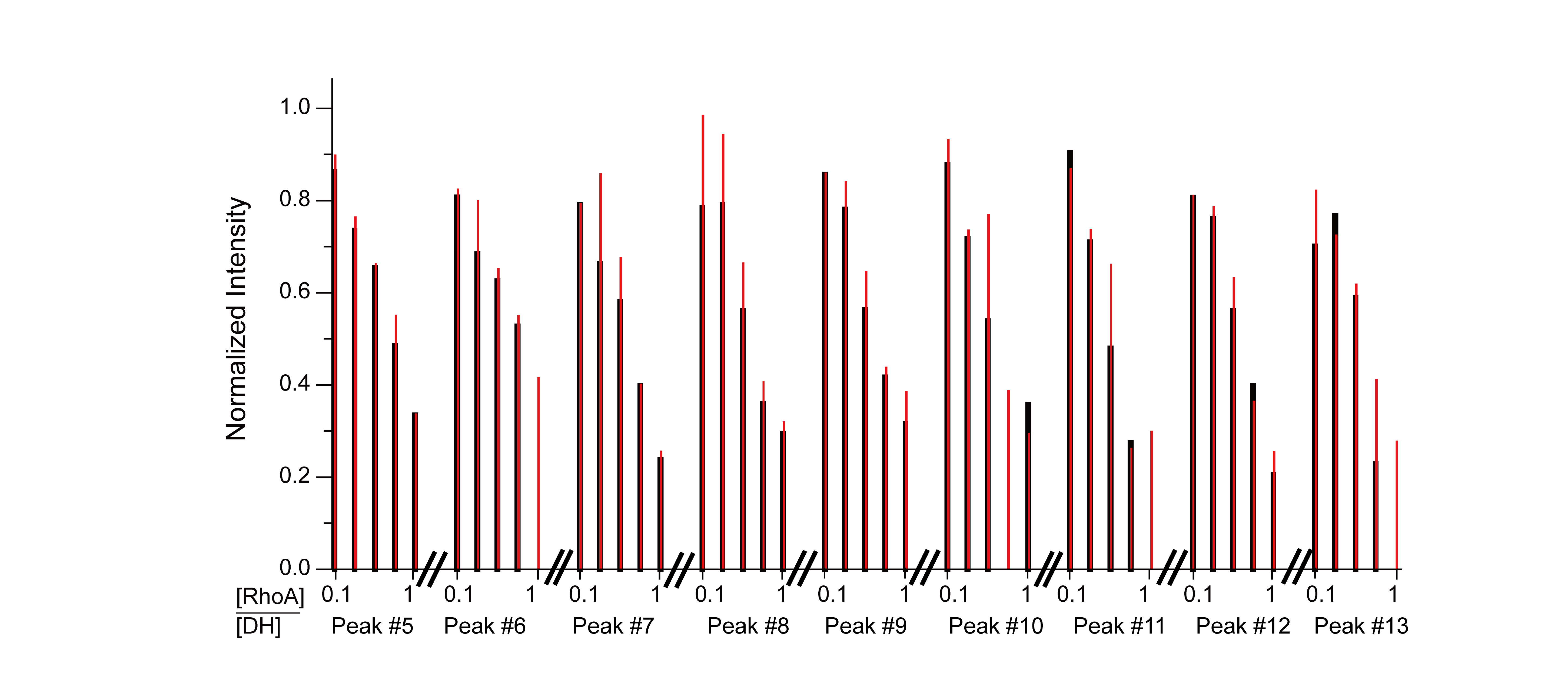

Supplement: Figure S6 — Correlation between peak intensities of 15N DH and RhoA/DH molar ratio, in the presence of DMSO (black lines) and compound R1 (red lines), respectively. Peaks 5–13 (numbered in Figure S5g) were separated by double lines. The intensities were normalized over those corresponding peaks in the absence of RhoA for each sample. Peaks with intensity less than 3 fold noise level were not measured. (TIF) [file pone.0088098.s006.tif]
