# Supplementary material for: Automated NMR Fragment Based Screening Identified a Novel Interface Blocker to the LARG/RhoA Complex
Source: PLoS One. 2014 Feb 5;9(2):e88098. doi: 10.1371/journal.pone.0088098 (PMC3914932; doi:10.1371/journal.pone.0088098)
Supplement: File S1 — This file includes Scripts S1–S6. Script S1.ACD/Automation script for filtration of commercial available fragment compounds based on the Rule of Three. Script S2. Automation script for the exclusion of compounds with a high Tanimoto similarity score in comparison with any existing member of the fragment library. Script S3. Script for thedetermination of the aqueous solubility concentration. Script S4. Upload the compounds with appropriate aqueous solubility and impurity levels to the final screening database. Script S5. Preparation of fragment cocktails with dispersed proton spectra. Script S6. Script for the processing and visualization of the fragment spectra of Watergate, STD and WaterLOGSY. (DOC) [file pone.0088098.s007.doc]

**Supporting Information**

**Automated NMR Fragment Based Screening Identified a Novel Interface Blocker to the LARG/RhoA Complex**

Jia Gao, Rongsheng Ma, Wei Wang, Na Wang, Ryan Sasaki, David Snyderman, Jihui Wu^1^, Ke Ruan^*^

Email: [kruan@ustc.edu.cn](mailto:kruan@ustc.edu.cn)

**Script S1.** ACD/Automation script for filtration of commercial available fragment compounds based on the Rule of Three.

/*

ACD/Automation Script features:

1. Filter the SDF based on the RO3: cLogP<3, 110<FW<350, Hacc<=3, Hdon<=3,RB<=3,logSw<=-4.5,tPSA<110;

2. Filter Molecules without aromatic protons.

Ke Ruan & Rongsheng Ma Copyright (c) 2011.

kruan@ustc.edu

*/

bool ReadPPMRange(string chkstr, double& Lowppm, double& Highppm)

{

int j;

chkstr=ExtractWord(2,chkstr,"[");

chkstr=ExtractWord(1,chkstr,"]");

j=Pos("..",chkstr);

Lowppm=StrToFloat(Trim(Copy(chkstr,1,j-1)));

Highppm=StrToFloat(Trim(Copy(chkstr,j+2,Length(chkstr)-j-1)));

return true;

}

bool DoCombinedVerification(TSpecDocument Doc1D, TSpecDocument DocHSQC, TSpecDocument& Doc1Dcalc, TSpecDocument& DocHSQCcalc)

{

TCombinedVerificationParams Params;

string ScriptPath;

ScriptPath = ExtractFilePath(GetScriptFileName);

Params=TCombinedVerificationParams.Create();

Params.C13_ShiftLooseness=10;

Params.C13_ShiftTolerance=1;

Params.C13_ShiftWeight=0.40;

Params.CalculationParams.C13_CalcMethod=scm_CorrectedWeightedAverage;

Params.CalculationParams.C13_UseDB=False;

//Params.CalculationParams.C13_UserDBs="";

Params.CalculationParams.H1_CalcExchangeJ=True;

Params.CalculationParams.H1_CalcMethod=scm_CorrectedWeightedAverage;

Params.CalculationParams.H1_IgnoreExchangeH=True;

Params.CalculationParams.H1_MergeExchangeSignals=False;

Params.CalculationParams.H1_Minimal_JValue=1.0;

Params.CalculationParams.H1_Use4JAndGreater=False;

Params.CalculationParams.H1_UseDB=False;

Params.CalculationParams.H1_UseMinimal_JValue=True;

//Params.CalculationParams.H1_UserDBs="";

Params.CalculationParams.H1_Use_HF_Consts=True;

Params.CalculationParams.H1_Use_HP_Consts=True;

Params.ConsistentAssignment=True;

Params.CreateCalcD1Doc=True;

Params.CreateCalcD2Doc=True;

Params.H1_ShiftLooseness=1;

Params.H1_ShiftTolerance=0.2;

Params.H1_ShiftWeight=0.30;

Params.IntegralAccuracy=0.15;

Params.LimitedOptimization=True;

Params.Minimal_J_Value=3.0;

Params.Multiplicity_Weight=0.15;

Params.QuantativeParameter=qpar_NucNumber;

Params.Quantitive_Weight=0.15;

Params.Update1DAssignmentsToDoc=True;

Params.Update2DAssignmentsToDoc=True;

if (CombinedVerification(Doc1D,DocHSQC,Doc1Dcalc,DocHSQCcalc,Params))

{

Params.Free();

return true;

}

else

{

Params.Free();

return false;

}

}

string ReadPar(string ProcparName, string ParName)

{

TStringList ParFile;

int i,j,count;

string chkstr;

ParFile=TStringList.Create();

if(!ParFile.LoadFromFile(ProcparName))

{

LogInfo("Error: can not open procpar file.");

return "";

}

i=0;

count=ParFile.Count();

while (i<count)

{

chkstr=ParFile[i];

j=Pos(" ",chkstr);

if (Trim(Left(chkstr,j))==ParName)

{

ParName = ExtractWord(2,ParFile[i+1],"\"");

if (ParName == "") ParName = ExtractWord(2,ParFile[i+1]," ");

break;

}

i++;

}

ParFile.Free();

return ParName;

}

String Lookup(String InitFileName, String ParName)

{

TStringList Setting;

int i,j,k,DimStr,DimDelim=4,flag;

string chkstr,Delim[4];

Setting=TStringList.Create();

Setting.LoadFromUnicodeFile(InitFileName);

DimStr=Setting.count;

flag=0;

k=Length(ParName);

for (i=0;i<DimStr;i++)

{

chkstr=Setting[i];

if(Trim(Left(chkstr,k))==ParName) {flag=1; break;}

}

Setting.Free();

if (flag==1) return Trim(Right(chkstr,Length(chkstr)-k));

else return "";

}

bool CalcHNum(TSpecDocument Doc1Dcalc,int& MinH)

{

TSpecmanTable TabMult;

string chkstr,ScriptPath;

double ILowCutOff, IHighCutOff,Lowppm[255],Highppm[255];

int i,itmp,rowcnt,IDppm,IDH;

Scriptpath = ExtractFilePath(GetScriptFileName);

Doc1Dcalc.SaveAs(ScriptPath+"testcalc.esp");

TabMult = Doc1Dcalc.GetTableByName("Table of Multiplets");

rowcnt = TabMult.RowsCount();

IDppm = TabMult.FindColumnIndex("(ppm)");

IDH = TabMult.FindColumnIndex("H's");

SetLength(Lowppm,rowcnt);

SetLength(Highppm,rowcnt);

chkstr = Lookup(ScriptPath+"Setting.txt","ILowCutOff");

if (chkstr != "") ILowCutOff = StrToFloat(chkstr);

else ILowCutOff = 0.0;

chkstr = Lookup(ScriptPath+"Setting.txt","IHighCutOff");

if (chkstr != "") IHighCutOff = StrToFloat(chkstr);

else IHighCutOff = 10.0;

MinH = 0;

for(i=0;i<rowcnt;i++)

{

itmp = StrToInt(TabMult.GetValue(i,IDH));

ReadPPMRange(TabMult.GetValue(i,IDppm),Lowppm[i],Highppm[i]);

if ( (Lowppm[i]>ILowCutOff) && (Highppm[i]<IHighCutoff) ) MinH += itmp;

}

TabMult.Free();

return true;

}

double RunQNMR(TChemicalStructure MolStructure)

{

TSpecDocument Doc,DocHSQC, Doc1DCalc, DocHSQCcalc;

TStringList MacCmd;

string chkstr, MacroRes,ScriptPath,IMacroName;

int MinH=1;

int i,j;

ScriptPath=ExtractFilePath(GetScriptFileName);

chkstr = Lookup(ScriptPath+"Setting.txt","IDummyHSQC");

if (Pos("\\",chkstr) == 0) chkstr = ScriptPath+chkstr;

DocHSQC = ImportDocument(chkstr);

DocHSQC.AddChemicalStructure(MolStructure);

chkstr = Lookup(ScriptPath+"Setting.txt","IDummyProton");

Doc = ImportDocument(chkstr);

Doc.AddChemicalStructure(MolStructure);

MacCMd = TStringList.Create();

IMacroName = Lookup(ScriptPath+"Setting.txt","IMacroName");

if(FileExists(IMacroName))

{

MacCmd.LoadFromFile(IMacroName);

j=MacCmd.Count();

for(i=0;i<j;i++) Doc.ProcessMacroCommand(MacCmd[i],MacroRes);

}

if (DoCombinedVerification(Doc,DocHSQC,Doc1Dcalc,DocHSQCcalc))

{

CalcHNum(Doc1Dcalc,MinH);

LogInfo(IntToStr(MinH));

}

// Doc1Dcalc.SaveAs(ScriptPath+"calctest.esp");

CloseDocument(Doc);

CloseDocument(DocHSQC);

CloseDocument(Doc1DCalc);

CloseDocument(DocHSQCcalc);

MacCmd.Free();

return MinH;

}

bool RO3Filter(string SDFFileName)

{

TStringList SDFList,TmpList,MolList,CheckList;

TChemicalStructure MolStructure;

string ScriptPath,OSDFFile,IRBName, IcLogPName,ItPSAName,IHaccName,IHdonName,ILogSwName,IFWName,IIDName;

double FRBMax, FcLogPMax,FtPSAMax,FHaccMax,FHdonMax,FLogSwMin,FMWMin,FMWMax;

double RB, cLogP,tPSA,Hacc,Hdon,LogSw,MW,aromh;

int i,j,k,count,count2,flag,ilen;

string chkstr,ID;

MolStructure=TChemicalStructure.Create();

ScriptPath=ExtractFilePath(GetScriptFileName);

OSDFFile = Lookup(ScriptPath+"Setting.txt","OSDFFile");

IRBName = Lookup(ScriptPath+"Setting.txt","IRBName");

IcLogPName = Lookup(ScriptPath+"Setting.txt","IcLogPName");

ItPSAName = Lookup(ScriptPath+"Setting.txt","ItPSAName");

IHaccName = Lookup(ScriptPath+"Setting.txt","IHaccName");

IHdonName = Lookup(ScriptPath+"Setting.txt","IHdonName");

ILogSwName = Lookup(ScriptPath+"Setting.txt","ILogSwName");

IFWName = Lookup(ScriptPath+"Setting.txt","IFWName");

IIDName = Lookup(ScriptPath+"Setting.txt","IIDName");

FRBMax = StrToFloat(Lookup(ScriptPath+"Setting.txt","FRBMax"));

FcLogPMax = StrToFloat(Lookup(ScriptPath+"Setting.txt","FcLogPMax"));

FtPSAMax = StrToFloat(Lookup(ScriptPath+"Setting.txt","FtPSAMax"));

FHaccMax = StrToFloat(Lookup(ScriptPath+"Setting.txt","FHaccMax"));

FHdonMax = StrToFloat(Lookup(ScriptPath+"Setting.txt","FHdonMax"));

FLogSwMin = StrToFloat(Lookup(ScriptPath+"Setting.txt","FLogSwMin"));

FMWMax = StrToFloat(Lookup(ScriptPath+"Setting.txt","FMWMax"));

FMWMin = StrToFloat(Lookup(ScriptPath+"Setting.txt","FMWMin"));

SDFList = TStringList.Create();

SDFList.LoadFromUnicodeFile(SDFFileName);

TmpList = TStringList.Create();

MolList = TStringList.Create();

CheckList = TStringList.Create();

count = SDFList.Count();

for(i=0;i<count;i++)

{

j=i;

while (Trim(SDFList[j]) != "M END")

{

MolList.Add(SDFList[j]);

j++;

}

MolList.Add("M END");

j=i;

while (Trim(SDFList[j]) != "$$$$")

{

TmpList.Add(SDFList[j]);

j++;

}

TmpList.Add("$$$$\n");

count2 = TmpList.Count();

for(k=0;k<count2;k++)

{

ilen=Length(IRBName);

if(Trim(Left(TmpList[k],ilen))==IRBName) RB = StrToFloat(TmpList[k+1]);

ilen=Length(IcLogPName);

if(Trim(Left(TmpList[k],ilen))==IcLogPName) cLogP = StrToFloat(TmpList[k+1]);

ilen=Length(ItPSAName);

if(Trim(Left(TmpList[k],ilen))==ItPSAName) tPSA = StrToFloat(TmpList[k+1]);

ilen=Length(IHaccName);

if(Trim(Left(TmpList[k],ilen))==IHaccName) Hacc = StrToFloat(TmpList[k+1]);

ilen=Length(IHdonName);

if(Trim(Left(TmpList[k],ilen))==IHdonName) Hdon = StrToFloat(TmpList[k+1]);

// ilen=Length(ILogSwName);

// if(Trim(Left(TmpList[k],ilen))==ILogSwName) LogSw = StrToFloat(TmpList[k+1]);

ilen=Length(IIDName);

if(Trim(Left(TmpList[k],ilen))==IIDName) ID = TmpList[k+1];

ilen=Length(IFWName);

if(Trim(Left(TmpList[k],ilen))==IFWName)

{

chkstr = TmpList[k+1];

if(Pos("(",chkstr)==0) MW = StrToFloat(chkstr);

else MW = StrToFloat(Copy(chkstr,Pos("(",chkstr)+1,Pos("+",chkstr)-Pos("(",chkstr)-1));

}

}

CheckList.Add("ID = "+ID+"; MW = "+FloatToStr(MW));

CheckList.AddToFile(ScriptPath+"MolLog.txt");

CheckList.Clear();

flag=0;

if (RB > FRBMax) flag=1;

if (cLogP > FcLogPMax) flag=1;

if (tPSA > FtPSAMax) flag=1;

if (Hacc > FHaccMax) flag=1;

if (Hdon > FHdonMax) flag=1;

// if (LogSw < FLogSwMin) flag=1;

if (MW > FMWMax) flag=1;

if (MW < FMWMin) flag=1;

aromh = 0.0;

if (flag==0)

{

MolList.SaveToFile(ScriptPath+"test1.mol");

MolStructure.LoadFromMolFile(ScriptPath+"test1.mol");

aromh = RunQNMR(MolStructure);

if (aromh >0)

{

TmpList.AddToFile(OSDFFile);

CheckList.Add("ID = "+ID+"; Arom H: "+FloatToStr(aromh));

CheckList.AddToFile(ScriptPath+"Filtered.txt");

CheckList.Clear();

}

}

TmpList.Clear();

MolList.Clear();

i=j+1;

}

SDFList.Free();

TmpList.Free();

MolList.Free();

CheckList.Free();

MolStructure.Free();

return true;

}

/************* Main script *********************/

{

RO3Filter(WorkFileName);

}

**Script S2.** Automation script for the exclusion of compounds with a high Tanimoto similarity score in comparison with any existing member of the fragment library.

/*

ACD/Automation Script features:

1. Filter the SDF based on Tanimoto similarity score.

Ke Ruan & Rongsheng Ma Copyright (c) 2011.

kruan@ustc.edu.cn

*/

String Lookup(String InitFileName, String ParName)

{

TStringList Setting;

int i,j,k,DimStr,DimDelim=4,flag;

string chkstr,Delim[4];

Setting=TStringList.Create();

Setting.LoadFromUnicodeFile(InitFileName);

DimStr=Setting.count;

flag=0;

k=Length(ParName);

for (i=0;i<DimStr;i++)

{

chkstr=Setting[i];

if(Trim(Left(chkstr,k))==ParName) {flag=1; break;}

}

Setting.Free();

if (flag==1) return Trim(Right(chkstr,Length(chkstr)-k));

else return "";

}

bool diversity(string SDFFileName)

{

TStringList SDFList,SDFList2,MolList,NameList,TmpList,chklist;

string ScriptPath,OSDFName,chkstr,OSDFFile;

int i,j,k,count,count3,count4,cntmax;

double tmax, FTanimoto;

ScriptPath=ExtractFilePath(GetScriptFileName);

FTanimoto = StrToFloat(Lookup(ScriptPath+"Setting.txt","FTanimoto"));

OSDFFile = Lookup(ScriptPath+"Setting.txt","OSDFFile");

SDFList = TStringList.Create();

SDFList.LoadFromUnicodeFile(SDFFileName);

SDFList2 = TStringList.Create();

SDFList2.LoadFromUnicodeFile(SDFFileName);

MolList = TStringList.Create();

TmpList = TStringList.Create();

NameList = TStringList.Create();

chklist = TStringList.Create();

count3 = 0;

count = SDFList.Count();

for(i=0;i<count;i++)

{

j=i;

while (Trim(SDFList[j]) != "M END")

{

MolList.Add(SDFList[j]);

j++;

}

MolList.Add("M END");

j=i;

while (Trim(SDFList[j]) != "$$$$")

{

TmpList.Add(SDFList[j]);

j++;

}

TmpList.Add("$$$$\n");

for(k=0;k<j-i+1;k++) SDFList2.Delete(0);

SDFList2.SaveToFile(ScriptPath+"temp.sdf");

MolList.SaveToFile(ScriptPath+"test.mol");

ExecApp2("\"C:\\Program Files\\OpenBabel-2.3.0\\babel\"",ScriptPath+"test.mol "+ScriptPath+"temp.sdf"+" -ofpt",NameList);

NameList.SaveToFile(ScriptPath+"Tanimoto"+IntToStr(count3)+".txt");

count4=0;

tmax=0.0;

for(k=0;k<NameList.Count();k++)

{

if(Pos("> Tanimoto from first mol =",NameList[k]) ==0 ) continue;

chkstr = Trim(StringReplace(NameList[k],"> Tanimoto from first mol =",""));

if(chkstr != "")

{

if( StrToFloat(chkstr) > tmax ) {tmax = StrToFloat(chkstr); cntmax = count4;}

count4++;

}

}

chklist.Add("Mol "+IntToStr(count3+1)+" to "+IntToStr(count3+cntmax+2)+": "+FloatToStr(tmax));

chklist.AddToFile(ScriptPath+"tmax.txt");

if ( tmax<FTanimoto )

{

TmpList.AddToFile(OSDFFile);

chklist.Clear();

chklist.Add(IntToStr(count3+1));

chklist.AddToFile(ScriptPath+"Pass.txt");

}

chklist.Clear();

MolList.Clear();

TmpList.Clear();

NameList.Clear();

count3++;

i=j;

}

SDFList.Free();

MolList.Free();

NameList.Free();

chklist.Free();

return true;

}

/************* Main script *********************/

{

diversity(WorkFileName);

}

**Script S3.** Script for the determination of the aqueous solubility concentration.

/*

ACD/Automation Script features:

1. File system independent. All settings will be loaded from Setting.txt file;

2. All input, output and judgement are controlled by parameters in Setting.txt;

with the first letter of I, O and F, respectively;

3. Attach structure from MOL or SDF files;

4. Read and process multiple FIDs automatically;

5. Run QNMR if required based on internal and/or external standard;

6. Search LC-MS to determine the MW.

7. Email users results of esp and html files if required;

8. Update to Chemical E Notebook database (SQL) for user browsing.

revisions:

Dec 18 2010:

Jan 04 2011: Verification based proton number estimation;

Jan 27 2011: automated QNMR in prodution at LJ;

Mar 10 2011: LC-MS integrated;

Apr 11 2011: Filename search using WildCard (unique filename);

Ke Ruan Copyright (c) 2010.

kruan@ustc.edu.cn

*/

String PlateName, MolName, EmailAddr, UserID, ExpName,FIDName; //reserved global variables.

double ReadAgilentMass(string rptfile,TChemicalStructure MolStructure)

{

TStringList Setting;

int i,j,count,DimStr;

double IMassError,IMassAdduct[255],MSmass, ExpMass;

string ScriptPath,chkstr,MassStr;

chkstr = MolStructure.Formula(true);

LogInfo("Formula: "+chkstr);

if (chkstr != "") MSmass = CalculateMonoisotopicMass(chkstr);

ScriptPath = ExtractFilePath(GetScriptFileName);

IMassError = StrToFloat(Lookup(ScriptPath+"Setting.txt","IMassError"));

chkstr = Lookup(ScriptPath+"Setting.txt","IMassAdduct");

count = WordCount(chkstr,";");

SetLength(IMassAdduct,count);

for(i=0;i<count;i++) IMassAdduct[i] = StrToFloat(ExtractWord(i+1,chkstr,";"));

Setting = TStringList.Create();

Setting.LoadFromUnicodeFile(rptfile);

DimStr = Setting.count;

for (i=0;i<DimStr;i++)

{

chkstr = Setting[i];

if (trim(chkstr) == "Time (MS) MS Area or Ion")

{

i++;

while (i<DimStr-1)

{

chkstr = Trim(Setting[i]);

MassStr = ExtractWord(WordCount(chkstr," ")-1,chkstr," ");

if (MassStr == "") {i++; continue;}

ExpMass = StrToFloat(MassStr);

for (j=0;j<count;j++)

{

if( abs(MSmass+IMassAdduct[j]-ExpMass) < IMassError)

{

Setting.Free();

return ExpMass-IMassAdduct[j];

}

}

i++;

}

break;

}

}

Setting.Free();

return 0.0;

}

double ReadMass(string rptfile,TChemicalStructure MolStructure)

{

TStringList Setting;

int i,j,count,DimStr;

double IMassError,IMassAdduct[255],MSmass, ExpMass;

string ScriptPath,chkstr;

chkstr = MolStructure.Formula(true);

LogInfo("Formula: "+chkstr);

if (chkstr != "") MSmass = CalculateMonoisotopicMass(chkstr);

LogInfo(FloatToStr(MSmass));

ScriptPath = ExtractFilePath(GetScriptFileName);

IMassError = StrToFloat(Lookup(ScriptPath+"Setting.txt","IMassError"));

chkstr = Lookup(ScriptPath+"Setting.txt","IMassAdduct");

count = WordCount(chkstr,";");

SetLength(IMassAdduct,count);

for(i=0;i<count;i++) IMassAdduct[i] = StrToFloat(ExtractWord(i+1,chkstr,";"));

Setting = TStringList.Create();

Setting.LoadFromUnicodeFile(rptfile);

DimStr = Setting.count;

for (i=0;i<DimStr;i++)

{

chkstr = Setting[i];

j = Pos(" ",chkstr);

if (j == 0) j = Pos(chr(9),chkstr);

if (j == 0) continue;

if (Trim(Left(chkstr,j-1)) != "BPM") continue;

chkstr = Trim(Right(chkstr,Length(chkstr)-j));

if (chkstr == "") continue;

LogInfo(chkstr);

ExpMass = StrToFloat(chkstr);

for (j=0;j<count;j++)

{

if( abs(MSmass+IMassAdduct[j]-ExpMass) < IMassError)

{

Setting.Free();

return ExpMass-IMassAdduct[j];

}

}

}

return 0.0;

Setting.Free();

}

//Copy raw data to a user defined directory to avoid ACD scanning over those files repeatedly.

bool CopyNMR(string FIDPath)

{

string OFIDPath,ScriptPath,chkstr,FileExt;

int i;

ScriptPath = ExtractFilePath(GetScriptFileName);

OFIDPath = Lookup(ScriptPath+"Setting.txt","OFIDPath");

OFIDPath = ReplacePar(OFIDPath);

i=1;

if (OFIDPath == "") return false;

if (!DirExists(OFIDPath))

{

CreateDir(OFIDPath,true);

RmDir(OFIDPath);

}

else

{

chkstr=OFIDPath;

FileExt = ExtractFileExt(OFIDPath);

while (DirExists(chkstr))

{

chkstr = StringReplace(OFIDPath,FileExt,"_0"+IntToStr(i)+FileExt);

i++;

}

OFIDPath=chkstr;

}

CopyDir(FIDPath,OFIDPath);

if(FileExists(OFIDPath+"\\fid")) RmDir(FIDPath);

return true;

}

//Update the esp file link to a Chemistry Database (oracle based, e.g., Dotmatics, etc.)

bool UpdateSQL(string OESPLink, string DocNum)

{

TSQLExecutor SQLCmd;

string ISQLConStr, ISQLUserName, ISQLPassword,INMRColName,IFormName,ISmpColName,INBColName;

string FRegCmpd,FUpdateSQL;

string ScriptPath,chkstr;

ScriptPath = ExtractFilePath(GetScriptFileName);

FUpdateSQL = Lookup(ScriptPath+"Setting.txt","FUpdateSQL"+DocNum);

FUpdateSQL = ReplacePar(FUpdateSQL);

if (pos(FUpdateSQL,PlateName) ==0 ) return false;

ISQLConStr = Lookup(ScriptPath+"Setting.txt","ISQLConStr");

ISQLUserName = Lookup(ScriptPath+"Setting.txt","ISQLUserName");

ISQLPassword = Lookup(ScriptPath+"Setting.txt","ISQLPassword");

// ISQLPassword = EncodePassword(ISQLPassword);

SQLCmd = TSQLExecutor.Create(ISQLConStr,ISQLUserName,ISQLPassword);

// NMR table in database contains at least three columns, Registered Compound Number, Notebook Number and NMR Data Link;

IFormName = Lookup(ScriptPath+"Setting.txt","IFormName");

ISmpColName = Lookup(ScriptPath+"Setting.txt","ISmpColName");

INBColName = Lookup(ScriptPath+"Setting.txt","INBColName");

INMRColName = Lookup(ScriptPath+"Setting.txt","INMRColName");

FRegCmpd = Lookup(ScriptPath+"Setting.txt","FRegCmpd");

if (pos(FRegCmpd,FIDName) ==0) //compound not registered yet, update notebook number;

{

chkstr = "INSERT INTO \""+ISQLUserName+"\".\""+IFormName+"\" ("+INBColName+", "+INMRColName+") VALUES ('"+FIDName+"', '"+OESPLink+"')";

SQLCmd.ExecSQL(chkstr);

}

else //compound registered already, update compound ID;

{

chkstr = "INSERT INTO \""+ISQLUserName+"\".\""+IFormName+"\" ("+ISmpColName+", "+INMRColName+") VALUES ('"+FIDName+"', '"+OESPLink+"')";

SQLCmd.ExecSQL(chkstr);

}

SQLCmd.Free();

return true;

}

//Email users with the esp file link, and archived into a htm file;

//Warning message is sent out if the measured concentration is out of the range for QC samples.

bool SendResult(TSpecDocument Doc,string& OESPLink, string FIDPath, string DocNum)

{

TStringList HTMContent;

string OHTMLink,IIntAccuracy,FSendResult;

string MailServer,ScriptPath,MailSub,MailBody,AdmEmail;

string conct,conce,conci,qcres,FileExt,chkstr;

int i;

ScriptPath = ExtractFilePath(GetScriptFileName);

MailServer = Lookup(ScriptPath+"Setting.txt","MailServer");

AdmEmail = Lookup(ScriptPath+"Setting.txt","AdmEmail");

OESPLink = Lookup(ScriptPath+"Setting.txt","OESPLink"+DocNum);

OESPLink = ReplacePar(OESPLink);

if (Pos("\\",OESPLink) == 0) OESPLink = FIDPath + OESPLink;

chkstr=OESPLink;

FileExt = ExtractFileExt(OESPLink);

i=1;

while (FileExists(chkstr))

{

chkstr = StringReplace(OESPLink,FileExt,"_0"+IntToStr(i)+FileExt);

i++;

}

OESPLink=chkstr;

Doc.Copy.SaveAs(OESPLink);

MailSub = "NMR data available for compound "+MolName+" in project "+PlateName;

MailBody = "\nClick the below link to access your NMR data (open by ACD/NMR Processor)";

MailBody = MailBody+"\n\n"+OESPLink+"\n";

OHTMLink = Lookup(ScriptPath+"Setting.txt","OHTMLink"+DocNum);

OHTMLink = ReplacePar(OHTMLink);

HTMContent = TStringList.Create();

HTMContent.Add("<head>");

HTMContent.Add("<title>ACD NMR Data</title>");

HTMContent.Add("</head>");

HTMContent.Add("<body lang=EN-US link=blue vlink=blue style='tab-interval:.5in'>");

HTMContent.Add("<p>"+MailSub+"</p>");

HTMContent.Add("<p>Click the below link to access your NMR data (open by ACD/NMR Processor)</p>");

HTMContent.Add("<p><a href=\"File:////"+OESPLink+"\">"+OESPLink+"</a></p>");

// QC data

conct = Lookup(ScriptPath+"Setting.txt",FIDName);

if(conct != "")

{

qcres="Fail";

HTMContent.Add("<p>QC data: Expected concentration = "+conct+" mM;</p>");

MailBody = MailBody+"\nQC data: Expected concentration = "+conct+" mM;\n";

IIntAccuracy = Lookup(ScriptPath+"Setting.txt","IIntAccuracy");

conce = Doc.GetUserData("Concentration (mM, External)","DOCUMENT");

if (conce != "")

{

HTMContent.Add("<p>Experimental concentration (mM, External) = "+conce+" mM;</p>");

MailBody = MailBody + "\nExperimental concentration (mM, External) = "+conce+" mM;\n";

if (StrToFloat(conct)>0.0) if ( abs(StrToFloat(conce)-StrToFloat(conct))/StrToFloat(conct) < StrToFloat(IIntAccuracy) ) qcres="Pass";

}

conci = Doc.GetUserData("Concentration (mM, Internal)","DOCUMENT");

if (conci != "")

{

HTMContent.Add("<p>Experimental concentration (mM, Internal) = "+conci+" mM;</p>");

MailBody = MailBody + "\nExperimental concentration (mM, Internal) = "+conci+" mM;\n";

if (StrToFloat(conct)>0.0) if ( abs(StrToFloat(conci)-StrToFloat(conct))/StrToFloat(conct) < StrToFloat(IIntAccuracy) ) qcres="Pass";

}

HTMContent.Add("<p>QC Result: "+qcres+"</p>");

MailBody = MailBody +"\nQC Result: "+qcres+"\n";

}

HTMContent.Add("<br></br>");

HTMContent.Add("</body>");

HTMContent.Add("</html>");

HTMContent.AddToFile(OHTMLink);

FSendResult = Lookup(ScriptPath+"Setting.txt","FSendResult"+DocNum);

FSendResult = ReplacePar(FSendResult);

// if ( (pos(FSendResult,PlateName) !=0) && (EmailAddr!="") ) SendMail(MailServer,EmailAddr,MailSub,MailBody,AdmEmail,25,20);

HTMContent.Free();

return true;

}

// retrieve the low and high ppm from the string at the format of [xxx.xx .. xxx.xx]

bool ReadPPMRange(string chkstr, double& Lowppm, double& Highppm)

{

int j;

chkstr=ExtractWord(2,chkstr,"[");

chkstr=ExtractWord(1,chkstr,"]");

j=Pos("..",chkstr);

Lowppm=StrToFloat(Trim(Copy(chkstr,1,j-1)));

Highppm=StrToFloat(Trim(Copy(chkstr,j+2,Length(chkstr)-j-1)));

return true;

}

bool DoCombinedVerification(TSpecDocument Doc1D, TSpecDocument DocHSQC, TSpecDocument& Doc1Dcalc, TSpecDocument& DocHSQCcalc)

{

TCombinedVerificationParams Params;

string ScriptPath;

ScriptPath = ExtractFilePath(GetScriptFileName);

Params=TCombinedVerificationParams.Create();

Params.C13_ShiftLooseness=10;

Params.C13_ShiftTolerance=1;

Params.C13_ShiftWeight=0.40;

Params.CalculationParams.C13_CalcMethod=scm_CorrectedWeightedAverage;

Params.CalculationParams.C13_UseDB=False;

//Params.CalculationParams.C13_UserDBs="";

Params.CalculationParams.H1_CalcExchangeJ=True;

Params.CalculationParams.H1_CalcMethod=scm_CorrectedWeightedAverage;

Params.CalculationParams.H1_IgnoreExchangeH=True;

Params.CalculationParams.H1_MergeExchangeSignals=False;

Params.CalculationParams.H1_Minimal_JValue=1.0;

Params.CalculationParams.H1_Use4JAndGreater=False;

Params.CalculationParams.H1_UseDB=False;

Params.CalculationParams.H1_UseMinimal_JValue=True;

//Params.CalculationParams.H1_UserDBs="";

Params.CalculationParams.H1_Use_HF_Consts=True;

Params.CalculationParams.H1_Use_HP_Consts=True;

Params.ConsistentAssignment=True;

Params.CreateCalcD1Doc=True;

Params.CreateCalcD2Doc=True;

Params.H1_ShiftLooseness=1;

Params.H1_ShiftTolerance=0.2;

Params.H1_ShiftWeight=0.30;

Params.IntegralAccuracy=0.15;

Params.LimitedOptimization=True;

Params.Minimal_J_Value=3.0;

Params.Multiplicity_Weight=0.15;

Params.QuantativeParameter=qpar_NucNumber;

Params.Quantitive_Weight=0.15;

Params.Update1DAssignmentsToDoc=True;

Params.Update2DAssignmentsToDoc=True;

if (CombinedVerification(Doc1D,DocHSQC,Doc1Dcalc,DocHSQCcalc,Params))

{

LogInfo("Combined Verification finished.");

Params.Free();

return true;

}

else

{

LogInfo("Combined Verification Failed!");

Params.Free();

return false;

}

}

bool CalcHNum(TSpecDocument Doc1Dcalc,int TotInt,int& MaxH, int& MinH)

{

TSpecmanTable TabMult;

string chkstr,ScriptPath;

double ILowCutOff, IHighCutOff,IHShiftTol,Lowppm[255],Highppm[255];

int i,itmp,rowcnt,IDppm,IDH,hcnt,hex;

Scriptpath = ExtractFilePath(GetScriptFileName);

TabMult = Doc1Dcalc.GetTableByName("Table of Multiplets");

rowcnt = TabMult.RowsCount();

IDppm = TabMult.FindColumnIndex("(ppm)");

IDH = TabMult.FindColumnIndex("H's");

SetLength(Lowppm,rowcnt);

SetLength(Highppm,rowcnt);

chkstr = Lookup(ScriptPath+"Setting.txt","ILowCutOff");

if (chkstr != "") ILowCutOff = StrToFloat(chkstr);

else ILowCutOff = 0.0;

chkstr = Lookup(ScriptPath+"Setting.txt","IHighCutOff");

if (chkstr != "") IHighCutOff = StrToFloat(chkstr);

else IHighCutOff = 10.0;

chkstr = Lookup(ScriptPath+"Setting.txt","IHShiftTol");

if (chkstr != "") IHShiftTol = StrToFloat(chkstr);

else IHShiftTol = 1.0;

MaxH = 0;

MinH = 0;

hcnt = 0;

for(i=0;i<rowcnt;i++)

{

itmp = StrToInt(TabMult.GetValue(i,IDH));

hcnt += itmp;

ReadPPMRange(TabMult.GetValue(i,IDppm),Lowppm[i],Highppm[i]);

if ( (Lowppm[i]>ILowCutOff-IHShiftTol) && (Highppm[i]<IHighCutoff+IHShiftTol) ) MaxH += itmp;

if ( (Lowppm[i]>ILowCutOff+IHShiftTol) && (Highppm[i]<IHighCutoff-IHShiftTol) ) MinH += itmp;

}

LogInfo("Nonexchangable Proton Number = "+IntToStr(hcnt));

hex = TotInt-hcnt;

MaxH += hex;

if (MinH<1) MinH = 1;

return true;

}

bool RunQNMR(TSpecDocument& Doc,string FIDPath,string DocNum,TChemicalStructure MolStructure)

{

TSpecmanTable TabInt;

TSpecDocument DocHSQC, DOC1DCalc, DocHSQCcalc;

string chkstr,ISolvent,ISystemName,FQNMR;

double ILowCutOff,IHighCutoff,IDefaultGain,IDefaultNT,IAmpFactor,IRefPeak,IRefConc,IImpLvl;

int IDefNumH;

string MacroRes,ScriptPath;

double tmp,tmp2,conc,nt,gain,IntRMSD,MinRMSD,IntAOI,TotInt,AbsAOI,AbsRef,Solppm[255];

double Lowppm[255],Highppm[255],Value[255];

int i,j,rowcnt,MinI,count,IDppm, IDvalue,IDabs,flag,MaxH,MinH;

conc=0.0;

ScriptPath = ExtractFilePath(GetScriptFileName);

FQNMR = Lookup(ScriptPath+"Setting.txt","FQNMR"+DocNum);

FQNMR = ReplacePar(FQNMR);

if (pos(FQNMR,PlateName) ==0 ) return false;

ISolvent = Lowercase(ReadPar(FIDPath+"procpar","solvent"));

chkstr = Lookup(ScriptPath+"Setting.txt","IRefPeak"+ISolvent);

IRefPeak = -20.0;

if (chkstr != "") IRefPeak = StrToFloat(chkstr); //read peak position of the external standard;

chkstr = Lookup(ScriptPath+"Setting.txt","IRefConc"+ISolvent);

IRefConc = -1.0;

if (chkstr != "") IRefConc = StrToFloat(chkstr); //read concentration of the external standard;

chkstr = Lookup(ScriptPath+"Setting.txt","IImpLvl");

IImpLvl = 0.4;

if (chkstr != "") IImpLvl = StrToFloat(chkstr); //read concentration of the external standard;

chkstr = Lookup(ScriptPath+"Setting.txt","IDefNumH");

IDefNumH = 20;

if (chkstr != "") IDefNumH = StrToInt(chkstr); //read concentration of the external standard;

chkstr = Lookup(ScriptPath+"Setting.txt",ISolvent);

count = WordCount(chkstr,";");

for(i=0;i<count;i++) Solppm[i] = StrToFloat(ExtractWord(i+1,chkstr,";"));

TabInt = Doc.GetTableByName("Table of Integrals");

rowcnt = TabInt.RowsCount();

SetLength(Lowppm,rowcnt);

SetLength(Highppm,rowcnt);

IDppm = TabInt.FindColumnIndex("(ppm)");

IDabs = TabInt.FindColumnIndex("Absolute Value");

//remove solvent peaks and reprocess the spectra

flag=0;

AbsRef=-1.0;

for(i=0;i<rowcnt;i++)

{

ReadPPMRange(TabInt.GetValue(i,IDppm),Lowppm[i],Highppm[i]);

if ( (Lowppm[i]<IRefPeak) && (IRefPeak<Highppm[i]) ) AbsRef = StrToFloat(TabInt.GetValue(i,IDabs));

for(j=0;j<count;j++)

{

// LogInfo(FloatToStr(Lowppm[i])+" < "+FloatToStr(Solppm[j])+" < "+FloatToStr(Highppm[i]));

if((Solppm[j]>Lowppm[i]) && (Solppm[j]<Highppm[i]))

{

Doc.ProcessMacroCommand("SetDarkRegion (Range = "+FloatToStr(Lowppm[i])+".."+FloatToStr(Highppm[i])+"; Description = \"Solvent\"; Active = True; Calculated = False)",MacroRes);

flag=1;

}

}

}

if (flag==1)

{

Doc.ProcessMacroCommand("Clear (What = \"Multiplets\"; Range = Full)",MacroRes);

Doc.ProcessMacroCommand("Clear (What = \"Integrals\"; Range = Full)",MacroRes);

Doc.ProcessMacroCommand("Clear (What = \"Peaks\"; Range = Full)",MacroRes);

Doc.ProcessMacroCommand("BaseLine (Range = Full; Method = \"Polynomial\"; Order = 4)",MacroRes);

Doc.ProcessMacroCommand("PeakPicking (Range = Full; NoiseFactor = 5; Threshold = \"SignalNoise\"; MinSN = 5; PosPeaks = True; NegPeaks = False; EqualPosition = False; UseDerivation = False)",MacroRes);

Doc.ProcessMacroCommand("BuildMultiplets (Nucleus = \"1H\"; PeakDistance = 0.8000; Coupling = 0.1000; MaxConst = 25.0000; UsePeakFit = False; IntegralThreshold = "+FloatToStr(IImpLvl)+"; NormalizationType = \"ProtonsInStructure\"; IgnoreLabileProtons = False; FixedNumberofProtons = False; MaxWidth = 120.0000; SeparateMaxConst = False; SeparateSymm = False; ResolveOverlapped = False)",MacroRes);

if (MolStructure.AtomsCount()<1) Doc.ProcessMacroCommand("BuildMultiplets (Nucleus = \"1H\"; PeakDistance = 0.8000; Coupling = 0.1000; MaxConst = 25.0000; UsePeakFit = False; IntegralThreshold = "+FloatToStr(IImpLvl)+"; NormalizationType = \"AssumedProtons\"; AssumedNumber = "+IntToStr(IDefNumH)+"; MaxWidth = 120.0000; SeparateMaxConst = False; SeparateSymm = False; ResolveOverlapped = False)",MacroRes);

}

TabInt = Doc.GetTableByName("Table of Integrals");

rowcnt = TabInt.RowsCount();

SetLength(Lowppm,rowcnt);

SetLength(Highppm,rowcnt);

IDppm = TabInt.FindColumnIndex("(ppm)");

//remove weaker solvent peaks not picked up at first.

flag=0;

for(i=0;i<rowcnt;i++)

{

ReadPPMRange(TabInt.GetValue(i,IDppm),Lowppm[i],Highppm[i]);

for(j=0;j<count;j++)

{

if((Solppm[j]>Lowppm[i]) && (Solppm[j]<Highppm[i]))

{

Doc.ProcessMacroCommand("SetDarkRegion (Range = "+FloatToStr(Lowppm[i])+".."+FloatToStr(Highppm[i])+"; Description = \"Solvent\"; Active = True; Calculated = False)",MacroRes);

flag=1;

}

}

}

if (flag==1)

{

Doc.ProcessMacroCommand("Clear (What = \"Multiplets\"; Range = Full)",MacroRes);

Doc.ProcessMacroCommand("Clear (What = \"Integrals\"; Range = Full)",MacroRes);

Doc.ProcessMacroCommand("Clear (What = \"Peaks\"; Range = Full)",MacroRes);

Doc.ProcessMacroCommand("PeakPicking (Range = Full; NoiseFactor = 5; Threshold = \"SignalNoise\"; MinSN = 5; PosPeaks = True; NegPeaks = False; EqualPosition = False; UseDerivation = False)",MacroRes);

Doc.ProcessMacroCommand("BuildMultiplets (Nucleus = \"1H\"; PeakDistance = 0.8000; Coupling = 0.1000; MaxConst = 25.0000; UsePeakFit = False; IntegralThreshold = "+FloatToStr(IImpLvl)+"; NormalizationType = \"ProtonsInStructure\"; IgnoreLabileProtons = False; FixedNumberofProtons = False; MaxWidth = 120.0000; SeparateMaxConst = False; SeparateSymm = False; ResolveOverlapped = False)",MacroRes);

if (MolStructure.AtomsCount()<1) Doc.ProcessMacroCommand("BuildMultiplets (Nucleus = \"1H\"; PeakDistance = 0.8000; Coupling = 0.1000; MaxConst = 25.0000; UsePeakFit = False; IntegralThreshold = "+FloatToStr(IImpLvl)+"; NormalizationType = \"AssumedProtons\"; AssumedNumber = "+IntToStr(IDefNumH)+"; MaxWidth = 120.0000; SeparateMaxConst = False; SeparateSymm = False; ResolveOverlapped = False)",MacroRes);

}

TabInt = Doc.GetTableByName("Table of Integrals");

rowcnt = TabInt.RowsCount();

SetLength(Lowppm,rowcnt);

SetLength(Highppm,rowcnt);

SetLength(Value,rowcnt);

IDppm = TabInt.FindColumnIndex("(ppm)");

IDvalue = TabInt.FindColumnIndex("Value");

IDabs = TabInt.FindColumnIndex("Absolute Value");

TotInt = 0.0; //Total number of protons;

IntAOI = 0.0; //number of protons in the area of interest;

count = 0;

AbsAOI = 0.0; //sum of absolute intensity in the area of interest;

chkstr = Lookup(ScriptPath+"Setting.txt","ILowCutOff");

if (chkstr != "") ILowCutOff = StrToFloat(chkstr);

else ILowCutOff = 0.0;

chkstr = Lookup(ScriptPath+"Setting.txt","IHighCutOff");

if (chkstr != "") IHighCutOff = StrToFloat(chkstr);

else IHighCutOff = 10.0;

LogInfo("Area of Interest: "+FloatToStr(ILowCutOff)+" ppm to "+FloatToStr(IHighCutOff)+" ppm.");

for(i=0;i<rowcnt;i++)

{

ReadPPMRange(TabInt.GetValue(i,IDppm),Lowppm[i],Highppm[i]);

TotInt += StrToFloat(TabInt.GetValue(i,IDvalue));

if( (Lowppm[i]>ILowCutOff) && (Highppm[i]<IHighCutOff) )

{

Value[count] = StrToFloat(TabInt.GetValue(i,IDvalue));

AbsAOI += StrToFloat(TabInt.GetValue(i,IDabs));

IntAOI += Value[count];

count++;

}

}

chkstr = Lookup(ScriptPath+"Setting.txt","IDummyHSQC");

chkstr = ReplacePar(chkstr);

if (Pos("\\",chkstr) == 0) chkstr = ScriptPath+chkstr;

DocHSQC = ImportDocument(chkstr);

DocHSQC.AddChemicalStructure(MolStructure);

MaxH = Round(TotInt);

MinH = 1;

Doc.SaveAs(ScriptPath+FIDName+".esp");

if (DoCombinedVerification(Doc,DocHSQC,Doc1Dcalc,DocHSQCcalc))

{

Doc = ImportDocument(ScriptPath+FIDName+".esp");

CalcHNum(Doc1Dcalc,Round(TotInt),MaxH,MinH);

}

DeleteFile(ScriptPath+FIDName+".esp");

LogInfo("Total Number of Protons = "+IntToStr(Round(TotInt)));

LogInfo("Max(H) = "+IntToStr(MaxH)+ " Min(H) = "+IntToStr(MinH));

MinRMSD=1e6;

// for(i=Round(TotInt);i>0;i--)

for(i=MinH;i<=MaxH;i++)

{

IntRMSD = 0.0;

for (j=0;j<count;j++)

{

tmp2 = Value[j]*i/IntAOI;

if ( tmp2-Int(tmp2) > Int(tmp2)+1.0-tmp2 ) tmp = Int(tmp2)+1.0-tmp2;

else tmp = tmp2-Int(tmp2);

if (MolStructure.AtomsCount()<1) IntRMSD += tmp*tmp;

else IntRMSD += abs(tmp);

}

if (MolStructure.AtomsCount()<1) IntRMSD = sqrt(IntRMSD/i);

else IntRMSD = IntRMSD/i;

if (IntRMSD < MinRMSD)

{

MinRMSD=IntRMSD;

MinI=i;

}

LogInfo("Integration Error at "+IntToStr(i)+" protons: "+FloatToStr(IntRMSD));

}

LogInfo("Abs Intensity = "+FloatToStr(AbsAOI)+"; Min(I) = "+IntToStr(MinI));

if (rowcnt >0)

{

if( MinI !=Round(IntAOI) )

{

Doc.ProcessMacroCommand("Clear (What = \"Multiplets\"; Range = Full)",MacroRes);

Doc.ProcessMacroCommand("Clear (What = \"Integrals\"; Range = Full)",MacroRes);

Doc.ProcessMacroCommand("Clear (What = \"Peaks\"; Range = Full)",MacroRes);

Doc.ProcessMacroCommand("PeakPicking (Range = Full; NoiseFactor = 5; Threshold = \"SignalNoise\"; MinSN = 5; PosPeaks = True; NegPeaks = False; EqualPosition = False; UseDerivation = False)",MacroRes);

Doc.ProcessMacroCommand("BuildMultiplets (Nucleus = \"1H\"; PeakDistance = 0.8000; Coupling = 0.1000; MaxConst = 25.0000; UsePeakFit = False; IntegralThreshold = "+FloatToStr(IImpLvl)+"; NormalizationType = \"AssumedProtons\"; AssumedNumber = "+IntToStr(Round(TotInt*MinI/IntAOI))+"; MaxWidth = 120.0000; SeparateMaxConst = False; SeparateSymm = False; ResolveOverlapped = False)",MacroRes);

}

chkstr = Lookup(ScriptPath+"Setting.txt","IDefaultGain");

if (chkstr != "") IDefaultGain = StrToFloat(chkstr);

else IDefaultGain = 48.0;

chkstr = Lookup(ScriptPath+"Setting.txt","IDefaultNT");

if (chkstr != "") IDefaultNT = StrToFloat(chkstr);

else IDefaultNT = 32;

ISystemName = Lookup(ScriptPath+"Setting.txt","ISystemName");

ISystemName = ReadPar(FIDPath+"procpar",ISystemName);

nt = StrToFloat(ReadPar(FIDPath+"procpar","nt"));

gain = StrToFloat(ReadPar(FIDPath+"procpar","gain"));

conc=AbsAOI*(IDefaultNT/nt)*Exp(Ln(10.0)*(IDefaultGain-gain)/20)/MinI;

LogInfo("IAmpFactor"+"."+ISystemName+"."+ExpName);

chkstr = Lookup(ScriptPath+"Setting.txt","IAmpFactor"+"."+ISystemName+"."+ExpName);

if (chkstr != "")

{

IAmpFactor = StrToFloat(chkstr);

Doc.ProcessMacroCommand("SetUserData (Name = \"Concentration (mM, External)\"; Value = \""+FloatToStr(conc/IAmpFactor)+"\"; Type = \"Spectrum\")",MacroRes);

if ((AbsRef >0.0)&& (IRefConc>0.0)) Doc.ProcessMacroCommand("SetUserData (Name = \"Concentration (mM, Internal)\"; Value = \""+FloatToStr(AbsAOI/MinI*IRefConc/AbsRef)+"\"; Type = \"Spectrum\")",MacroRes);

}

else LogInfo("Amplification factor not found. QNMR exit...");

}

else Doc.ProcessMacroCommand("SetUserData (Name = \"Concentration (mM)\"; Value = \"NA\"; Type = \"Spectrum\")",MacroRes);

TabInt.Free();

return true;

}

bool ProcessDoc(TSpecDocument Doc, string FIDPath,string DocNum, TChemicalStructure MolStructure)

{

TStringList MacCmd;

double IRefPeak;

string IMacroName,ISolvent,FAutoRef,FAutoSync,FQNMR;

string MacroRes,ScriptPath,chkstr;

int np,ni,i,j;

if (Doc == nil) return false;

ScriptPath=ExtractFilePath(GetScriptFileName);

Doc.AddChemicalStructure(MolStructure);

IMacroName = Lookup(ScriptPath+"Setting.txt","IMacroName"+DocNum);

IMacroName = ReplacePar(IMacroName);

if (Pos("\\",IMacroName) ==0 ) IMacroName=ScriptPath+IMacroName;

chkstr = ReadPar(FIDPath+"procpar","page");

Doc.ProcessMacroCommand("SetUserData (Name = \"Page\"; Value = \""+chkstr+"\"; Type = \"Spectrum\")",MacroRes);

Doc.ProcessMacroCommand("SetUserData (Name = \"User ID\"; Value = \""+UserID+"\"; Type = \"Spectrum\")",MacroRes);

Doc.ProcessMacroCommand("SetUserData (Name = \"Email Address\"; Value = \""+EmailAddr+"\"; Type = \"Spectrum\")",MacroRes);

Doc.ProcessMacroCommand("SetUserData (Name = \"Project Name\"; Value = \""+PlateName+"\"; Type = \"Spectrum\")",MacroRes);

Doc.ProcessMacroCommand("SetUserData (Name = \"Sample Name\"; Value = \""+FIDName+"\"; Type = \"Spectrum\")",MacroRes);

Doc.ProcessMacroCommand("SetUserData (Name = \"Structure Name\"; Value = \""+MolName+"\"; Type = \"Spectrum\")",MacroRes);

Doc.ProcessMacroCommand("SetUserData (Name = \"Experimental Name\"; Value = \""+ExpName+"\"; Type = \"Spectrum\")",MacroRes);

Doc.ProcessMacroCommand("SetUserData (Name = \"Verification\"; Value = \" \"; Type = \"Spectrum\")",MacroRes);

MacCmd=TStringList.Create();

chkstr = Doc.GetParameter("Original Points Count");

if (Pos("(",chkstr)==0) //1D NMR

{

np = StrToInt(chkstr);

np = np*2;

i=1024;

while (i<np) i=i*2;

Doc.ProcessMacroCommand("ZeroFilling (PointsCount = \""+IntToStr(i)+"\")",MacroRes);

}

if(FileExists(IMacroName))

{

MacCmd.LoadFromFile(IMacroName);

j=MacCmd.Count();

for(i=0;i<j;i++) Doc.ProcessMacroCommand(MacCmd[i],MacroRes);

}

if (MolStructure.AtomsCount()<1)

{

FQNMR = Lookup(ScriptPath+"Setting.txt","FQNMR"+DocNum);

FQNMR = ReplacePar(FQNMR);

if (pos(FQNMR,PlateName) !=0 ) Doc.ProcessMacroCommand("BuildMultiplets (Nucleus = \"1H\"; PeakDistance = 0.8000; Coupling = 0.1000; MaxConst = 25.0000; UsePeakFit = False; IntegralThreshold = 0.1000; NormalizationType = \"AssumedProtons\"; AssumedNumber = 40; MaxWidth = 120.0000; SeparateMaxConst = False; SeparateSymm = False; ResolveOverlapped = False)",MacroRes);

}

//Set reference based on solvent peak;

ISolvent = ReadPar(FIDPath+"procpar","solvent");

chkstr = Lookup(ScriptPath+"Setting.txt","IRefPeak"+ISolvent);

IRefPeak = -20.0;

if (chkstr != "") IRefPeak = StrToFloat(chkstr); //read peak position of the external standard;

FAutoRef = Lookup(ScriptPath+"Setting.txt","FAutoRef"+DocNum);

if(PlateName=="SNQCOMIC1J") Doc.ProcessMacroCommand("Reference (OldPosition = 3.50; NewPosition = 2.50; Name = \""+ISolvent+"\")",MacroRes);

if ( (Lowercase(FAutoRef) =="y") || (Lowercase(FAutoRef) == "yes") )

{

Doc.ProcessMacroCommand("FindPeak (Position = "+FloatToStr(IRefPeak)+"; Tolerance = 0.5; Property = \"AbsHeight\"; Criteria = \"Maximal\"; Range = 0.0000..0.0000; Value = 0.0000; IgnoreAnnotated = False; Result = Pos)",MacroRes);

if (MacroRes=="") continue;

MacCmd.Clear();

MacCmd.Add(FIDName+" "+FloatToStr(IRefPeak-StrToFloat(MacroRes)));

Doc.ProcessMacroCommand("Reference (OldPosition = "+MacroRes+"; NewPosition = "+FloatToStr(IRefPeak)+"; Name = \""+ISolvent+"\")",MacroRes);

MacCmd.SaveToFile(ScriptPath+"RefSync.txt");

}

//Synchronize over other spectra;

FAutoSync = Lookup(ScriptPath+"Setting.txt","FAutoSync"+DocNum);

if ( (Lowercase(FAutoSync) =="y") || (Lowercase(FAutoSync) == "yes") )

{

chkstr = Lookup(ScriptPath+"RefSync.txt",FIDName);

if (chkstr == "")

{

DeleteFile(ScriptPath+"RefSync.txt");

continue;

}

Doc.ProcessMacroCommand("Reference (OldPosition = 0.0; NewPosition = "+chkstr+"; Name = \"Synchronize\")",MacroRes);

}

MacCmd.Free();

return true;

}

//Replace wildcard in the filename

string ReplaceWildCard(string FileName)

{

TStringList NameList;

string tmpname,chkstr;

int i,count;

if (Pos("*",FileName)==0) return FileName;

NameList = TStringList.Create();

count = WordCount(FileName,"\\");

chkstr = "";

for(i=1;i<count;i++)

{

tmpname = ExtractWord(i,FileName,"\\");

if (Pos("*",tmpname)==0) chkstr = chkstr+tmpname+"\\";

else

{

ExecApp2("CMD","/C DIR /B "+chkstr+tmpname,NameList);

if(NameList.Count() ==1) chkstr = chkstr + NameList[0]+"\\";

else

{

NameList.Free();

LogInfo("FileName Incorrect!");

return "";

}

}

}

tmpname = ExtractWord(count,FileName,"\\");

if (Pos("*",tmpname)==0) FileName = chkstr+tmpname;

else

{

ExecApp2("CMD","/C DIR /B "+chkstr+tmpname,NameList);

if(NameList.Count() ==1) FileName = chkstr + NameList[0];

else

{

NameList.Free();

LogInfo("FileName Incorrect!");

return "";

}

}

NameList.Free();

return FileName;

}

string ReplacePar(string ParName)

{

string chkstr;

chkstr = StringReplace(ParName,"ParPlateName",PlateName);

chkstr = StringReplace(chkstr,"ParMolName",MolName);

chkstr = StringReplace(chkstr,"ParEmailAddr",EmailAddr);

chkstr = StringReplace(chkstr,"ParUserID",UserID);

chkstr = StringReplace(chkstr,"ParExpName",ExpName);

chkstr = StringReplace(chkstr,"ParFIDName",FIDName);

return chkstr;

}

string ReadPar(string ProcparName, string ParName)

{

TStringList ParFile;

int i,j,count;

string chkstr;

ParFile=TStringList.Create();

if(!ParFile.LoadFromFile(ProcparName))

{

LogInfo("Error: can not open procpar file.");

ParFile.Free();

return "";

}

i=0;

count=ParFile.Count();

while (i<count)

{

chkstr=ParFile[i];

j=Pos(" ",chkstr);

if (Trim(Left(chkstr,j))==ParName)

{

ParName = ExtractWord(2,ParFile[i+1],"\"");

if (ParName == "") ParName = ExtractWord(2,ParFile[i+1]," ");

break;

}

i++;

}

ParFile.Free();

return ParName;

}

String Lookup(String InitFileName, String ParName)

{

TStringList Setting;

int i,j,k,DimStr,DimDelim=4,flag;

string chkstr,Delim[4];

Setting=TStringList.Create();

Setting.LoadFromUnicodeFile(InitFileName);

DimStr=Setting.count;

flag=0;

k=Length(ParName);

for (i=0;i<DimStr;i++)

{

chkstr=Setting[i];

if(Trim(Left(chkstr,k))==ParName) {flag=1; break;}

}

Setting.Free();

if (flag==1) return Trim(Right(chkstr,Length(chkstr)-k));

else return "";

}

bool InitSetting(String FIDPath)

{

string ScriptPath;

//Load 5 global variables;

PlateName = "";

EmailAddr = "";

UserID = "";

MolName = "";

FIDName = "";

ExpName = "";

ScriptPath=ExtractFilePath(GetScriptFileName);

PlateName = Lookup(ScriptPath+"Setting.txt","ParPlateName");

EmailAddr = Lookup(ScriptPath+"Setting.txt","ParEmailAddr");

UserID = Lookup(ScriptPath+"Setting.txt","ParUserID");

MolName = Lookup(ScriptPath+"Setting.txt","ParMolName");

FIDName = Lookup(ScriptPath+"Setting.txt","ParFIDName");

ExpName = Lookup(ScriptPath+"Setting.txt","ParExpName");

//enumerate the global variable from procpar

if (!FileExists(FIDPath+"procpar"))

{

LogInfo("Error: procpar file not found.");

return false;

}

if(PlateName!="") PlateName = ReadPar(FIDPath+"procpar",PlateName);

if(UserID!="") UserID = ReadPar(FIDPath+"procpar",UserID);

if(MolName!="") MolName = ReadPar(FIDPath+"procpar",MolName);

if(FIDName!="") FIDName = ReadPar(FIDPath+"procpar",FIDName);

if(ExpName!="") ExpName = ReadPar(FIDPath+"procpar",ExpName);

if(EmailAddr!="")

{

EmailAddr = ReadPar(FIDPath+"procpar",EmailAddr);

EmailAddr = StringReplace(EmailAddr,"_",".");

}

return true;

}

/************* Main script *********************/

{

TSpecDocument Doc1,Doc2,Doc3,Doc4,Doc5;

TChemicalStructure MolStructure;

TSDFile SDFStructure;

TSpecManagerDatabase LocalDB;

string FMonitorFID,ODBPath,OCopyMol,OESPLink;

string IMolPath,IMassFile,ISDFPath,IFIDName,ISDFCompoundName;

string FIDPath,ScriptPath,MacroRes;

int SDIndex,RecID;

double tmp;

FIDPath=ExtractFilePath(WorkFileName);

// LogInfo(FIDPath);

if(!InitSetting(FIDPath))

{

LogInfo("Initialization Failed!");

exit;

}

ScriptPath=ExtractFilePath(GetScriptFileName);

FMonitorFID = Lookup(ScriptPath+"Setting.txt","FMonitorFID");

FMonitorFID = ReplacePar(FMonitorFID);

if (FMonitorFID != ExpName)

{

LogInfo("Error: Inappropriate 1D File Name!");

exit;

}

MolStructure=TChemicalStructure.Create();

IMolPath = Lookup(ScriptPath+"Setting.txt","IMolPath");

IMolPath = ReplacePar(IMolPath);

if (Pos("\\",IMolPath) == 0) IMolPath = FIDPath+IMolPath;

IMolPath=StringReplace(IMolPath,"PXP_","");

IMolPath = ReplaceWildCard(IMolPath);

if(FileExists(IMolPath)) MolStructure.LoadFromMolFile(IMolPath);

else

{

ISDFPath = Lookup(ScriptPath+"Setting.txt","ISDFPath");

ISDFPath = ReplacePar(ISDFPath);

ISDFPath = ReplaceWildCard(ISDFPath);

if (FileExists(ISDFPath))

{

SDFStructure = TSDFile.Open(ISDFPath,false);

ISDFCompoundName = Lookup(ScriptPath+"Setting.txt","ISDFCompoundName");

SDFStructure.FindIndexByData(ISDFCompoundName,MolName,SDIndex,false,0);

if (SDIndex >-1) MolStructure = SDFStructure.ReadStructure(SDIndex);

}

if (MolStructure.AtomsCount()<1)

{

ISDFPath = Lookup(ScriptPath+"Setting.txt","IGlobalSDF");

ISDFPath = ReplacePar(ISDFPath);

ISDFPath = ReplaceWildCard(ISDFPath);

if (FileExists(ISDFPath))

{

SDFStructure = TSDFile.Open(ISDFPath,false);

ISDFCompoundName = Lookup(ScriptPath+"Setting.txt","ISDFCompoundName");

SDFStructure.FindIndexByData(ISDFCompoundName,MolName,SDIndex,false,0);

if (SDIndex >-1) MolStructure = SDFStructure.ReadStructure(SDIndex);

}

}

}

/* process first spectrum */

Doc1=ImportDocument(WorkFileName);

FIDPath = ExtractFilePath(WorkFileName);

ProcessDoc(Doc1,FIDPath,"1",MolStructure);

RunQNMR(Doc1,FIDPath,"1",MolStructure);

OCopyMol = Lookup(ScriptPath+"Setting.txt","OCopyMol1");

if (OCopyMol != "")

{

OCopyMol = ReplacePar(OCopyMol);

if (Pos("\\",OCopyMol) == 0) OCopyMol = FIDPath+OCopyMol;

MolStructure.SaveToMolFile(OCopyMol);

}

CopyNMR(FIDPath);

SendResult(Doc1,OESPLink,FIDPath,"1");

// UpdateSQL(OESPLink,"1");

/* Process second spectrum */

/*

IFIDName = Lookup(ScriptPath+"Setting.txt","IFIDName2");

IFIDName = Replacepar(IFIDName);

IFIDName = ReplaceWildCard(IFIDName);

if ( FileExists(IFIDName) )

{

FIDPath = ExtractFilePath(IFIDName);

if(!InitSetting(FIDPath))

{

LogInfo("Initialization Failed!");

exit;

}

Doc2=ImportDocument(IFIDName);

ProcessDoc(Doc2,FIDPath,"2",MolStructure);

RunQNMR(Doc2,FIDPath,"2",MolStructure);

OCopyMol = Lookup(ScriptPath+"Setting.txt","OCopyMol2");

if (OCopyMol != "")

{

OCopyMol = ReplacePar(OCopyMol);

if ( Pos("\\",OCopyMol) ==0) OCopyMol = FIDPath+OCopyMol;

MolStructure.SaveToMolFile(OCopyMol);

}

CopyNMR(FIDPath);

SendResult(Doc2,OESPLink,FIDPath,"2");

UpdateSQL(OESPLink,"2");

}

*/

/* Process third spectrum */

/*

IFIDName = Lookup(ScriptPath+"Setting.txt","IFIDName3");

IFIDName = Replacepar(IFIDName);

if ( FileExists(IFIDName) )

{

FIDPath = ExtractFilePath(IFIDName);

if(!InitSetting(FIDPath))

{

LogInfo("Initialization Failed!");

exit;

}

Doc3=ImportDocument(IFIDName);

ProcessDoc(Doc3,FIDPath,"3",MolStructure);

RunQNMR(Doc3,FIDPath,"3",MolStructure);

OCopyMol = Lookup(ScriptPath+"Setting.txt","OCopyMol3");

if (OCopyMol != "")

{

OCopyMol = ReplacePar(OCopyMol);

if ( Pos("\\",OCopyMol) ==0) OCopyMol = FIDPath+OCopyMol;

MolStructure.SaveToMolFile(OCopyMol);

}

CopyNMR(FIDPath);

SendResult(Doc1,OESPLink,FIDPath,"3");

UpdateSQL(OESPLink,"3");

}

*/

/*

ProcessDoc(Doc4,FIDPath,"4",MolStructure);

ProcessDoc(Doc5,FIDPath,"5",MolStructure);

*/

//Process Mass spectrum in rpt format

IMassFile = Lookup(ScriptPath+"Setting.txt","IMassFile");

IMassFile = ReplacePar(IMassFile);

IMassFile = ReplaceWildCard(IMassFile);

if ( FileExists(IMassFile))

{

tmp = ReadMass(IMassFile,MolStructure);

LogInfo(FloatToStr(tmp));

if (tmp > 0.0) Doc1.ProcessMacroCommand("SetUserData (Name = \"Mass\"; Value = \""+FloatToStr(tmp)+"\"; Type = \"Spectrum\")",MacroRes);

else Doc1.ProcessMacroCommand("SetUserData (Name = \"Mass\"; Value = \"Not Found\"; Type = \"Spectrum\")",MacroRes);

}

//update the spectra to ACD database;

RecID=-1;

ODBPath=Lookup(ScriptPath+"Setting.txt","ODBPath");

ODBPath=ReplacePar(ODBPath);

LogInfo("OutPut Database: "+ODBPath);

LocalDB=TSpecManagerDatabase.OpenLocal(ODBPath,true,"","");

// LocalDB.UpdateSpecDocument2(Doc1,RecID);

// if (Doc2 != nil) LocalDB.UpdateSpecDocument2(Doc2,RecID);

// if (Doc3 != nil) LocalDB.UpdateSpecDocument2(Doc3,RecID);

// if (Doc4 != nil) LocalDB.UpdateSpecDocument2(Doc4,RecID);

// if (Doc5 != nil) LocalDB.UpdateSpecDocument2(Doc5,RecID);

MolStructure.Free();

if(SDFStructure != nil) SDFStructure.Free();

CloseDocument(Doc1);

if (Doc2!= nil) CloseDocument(Doc2);

if (Doc3 != nil) CloseDocument(Doc3);

if (Doc4 != nil) CloseDocument(Doc4);

if (Doc5 != nil) CloseDocument(Doc5);

LocalDB.Free();

}

*Macros for the processing of 1-D proton spectra.*

SetUserData (Name = "DOCDIR"; Value = "$(DOCDIR)"; Type = "Spectrum")

WindowFunction (Method = "Exponential"; LB = 0.5000)

FT (Operation = "Default")

Phase (Method = "Simple"; EqualPhase = False; FixPh1 = False)

BaseLine (Range = Full; Method = "Polynomial"; Order = 4)

PeakPicking (Range = Full; NoiseFactor = 5; Threshold = "SignalNoise"; MinSN = 5; PosPeaks = True; NegPeaks = False; EqualPosition = False; UseDerivation = False)

**Script S4.** Upload the compounds with appropriate aqueous solubility and impurity levels to the final screening database.

/*

ACD/Automation Script features:

1. Recalibrate concentration based on the user revised integrals;

2. Update esp files for fragment library to ACD nd9 database with acceptable concentration and consistent structure.

Ke Ruan Copyright (c) 2012.

rankly@hotmail.com

*/

String Lookup(String InitFileName, String ParName)

{

TStringList Setting;

int i,j,k,DimStr,DimDelim=4,flag;

string chkstr,Delim[4];

Setting=TStringList.Create();

Setting.LoadFromFile(InitFileName);

DimStr=Setting.count;

Delim[0]=",";

Delim[1]=" ";

Delim[2]=chr(9);

Delim[3]=";";

flag=0;

for (i=0;i<DimStr;i++)

{

chkstr=Setting[i];

k=-1;

for(j=0;j<DimDelim;j++)

{

k = Pos(Delim[j],chkstr);

if (k>0) break;

}

if (k>0) if (Trim(Left(chkstr,k-1))==ParName) {flag=1; break;}

}

Setting.Free();

if (flag==1) return Trim(Right(chkstr,Length(chkstr)-k));

else return "";

}

bool ReadPPMRange(string chkstr, double& Lowppm, double& Highppm)

{

int j;

chkstr=ExtractWord(2,chkstr,"[");

chkstr=ExtractWord(1,chkstr,"]");

j=Pos("..",chkstr);

Lowppm=StrToFloat(Trim(Copy(chkstr,1,j-1)));

Highppm=StrToFloat(Trim(Copy(chkstr,j+2,Length(chkstr)-j-1)));

return true;

}

bool Recalconc(TSpecDocument& Doc)

{

TSpecmanTable TabInt;

string chkstr,ScriptPath,MacroRes;

double ILowCutOff,IHighCutoff,IAmpFactor;

double conc,IntAOI,AbsAOI;

double Lowppm[255],Highppm[255];

int i,rowcnt,IDppm, IDvalue,IDabs;

ScriptPath = ExtractFilePath(GetScriptFileName);

TabInt = Doc.GetTableByName("Table of Integrals");

rowcnt = TabInt.RowsCount();

SetLength(Lowppm,rowcnt);

SetLength(Highppm,rowcnt);

IDppm = TabInt.FindColumnIndex("(ppm)");

IDabs = TabInt.FindColumnIndex("Absolute Value");

IDvalue = TabInt.FindColumnIndex("Value");

IntAOI = 0.0; //number of protons in the area of interest;

AbsAOI = 0.0; //sum of absolute intensity in the area of interest;

chkstr = Lookup(ScriptPath+"Setting.txt","ILowCutOff");

if (chkstr != "") ILowCutOff = StrToFloat(chkstr);

else ILowCutOff = 0.0;

chkstr = Lookup(ScriptPath+"Setting.txt","IHighCutOff");

if (chkstr != "") IHighCutOff = StrToFloat(chkstr);

else IHighCutOff = 10.0;

chkstr = Lookup(ScriptPath+"Setting.txt","IAmpFactor");

if (chkstr != "") IAmpFactor = StrToFloat(chkstr);

LogInfo("Area of Interest: "+FloatToStr(ILowCutOff)+" ppm to "+FloatToStr(IHighCutOff)+" ppm.");

for(i=0;i<rowcnt;i++)

{

ReadPPMRange(TabInt.GetValue(i,IDppm),Lowppm[i],Highppm[i]);

if( (Lowppm[i]>ILowCutOff) && (Highppm[i]<IHighCutOff) )

{

IntAOI = StrToFloat(TabInt.GetValue(i,IDvalue));

AbsAOI = StrToFloat(TabInt.GetValue(i,IDabs));

conc = AbsAOI/(IntAOI*IAmpFactor);

Doc.ProcessMacroCommand("SetUserData (Name = \"Concentration (mM, recal)\"; Value = \""+FloatToStr(conc)+"\"; Type = \"Spectrum\")",MacroRes);

break;

}

}

TabInt.Free();

return true;

}

/************* Main script *********************/

{

TSpecDocument Doc1;

TSpecManagerDatabase LocalDB;

string FMonitorFID,ODBPath,OESPFailed,FQNMR,FVerification,OESPFile;

string ScriptPath,ExpName,FIDPath,chkstr;

int RecID;

FIDPath = ExtractFilePath(WorkFileName);

LogInfo(WorkFileName);

Doc1 = ImportDocument(WorkFileName);

ExpName = Doc1.GetUserData("Experimental Name","DOCUMENT");

ScriptPath=ExtractFilePath(GetScriptFileName);

FVerification = Lookup(ScriptPath+"Setting.txt","FVerification");

chkstr = Doc1.GetUserData("Verification","DOCUMENT");

OESPFailed = Lookup(ScriptPath+"Setting.txt","OESPFailed");

OESPFile = ExtractFileName(WorkFileName);

if( (Trim(Lowercase(chkstr)) != Trim(Lowercase(FVerification))) && (Trim(Lowercase(chkstr)) !="") )

{

Doc1.Copy.SaveAs(OESPFailed+OESPFile);

CloseDocument(Doc1);

exit;

}

Recalconc(Doc1);

chkstr = Doc1.GetUserData("Concentration (mM, recal)","DOCUMENT");

FQNMR = Lookup(ScriptPath+"Setting.txt","FQNMR");

if (chkstr == "")

{

Doc1.Copy.SaveAs(OESPFailed+OESPFile);

CloseDocument(Doc1);

exit;

}

if (FQNMR != "")

{

if (StrToFloat(chkstr)<StrToFloat(FQNMR))

{

Doc1.Copy.SaveAs(OESPFailed+OESPFile);

CloseDocument(Doc1);

exit;

}

}

Doc1.Copy.SaveAs(Lookup(ScriptPath+"Setting.txt","OESPPath")+OESPFile);

RecID=-1;

ODBPath=Lookup(ScriptPath+"Setting.txt","ODBPath");

if (Pos("\\",ODBPath) == 0) ODBPath = FIDPath+ODBPath;

LocalDB=TSpecManagerDatabase.OpenLocal(ODBPath,true,"","");

LocalDB.UpdateSpecDocument2(Doc1,RecID);

CloseDocument(Doc1);

LocalDB.Free();

}

**Script S5.** Preparation of fragment cocktails with dispersed proton spectra.

/*

Optimization of fragment mixture preparation;

Ke Ruan Copyright (c) 2010.

rankly@hotmail.com

*/

String ExpName; //reserved global variables.

double GetOvlpLvl(string PeakList1,string PeakList2)

{

int i,j,PeakCnt1,PeakCnt2,OvlpCnt;

double Lowcs1,Upcs1,Lowcs2,Upcs2,OvlpLvl;

string Peak1,Peak2;

PeakCnt1 = WordCount(PeakList1,";");

PeakCnt2 = WordCount(PeakList2,";");

OvlpCnt = 0;

for (i=1;i<PeakCnt1;i++)

{

Peak1 = ExtractWord(i,PeakList1,";");

ReadPPMRange(Peak1,Lowcs1,Upcs1);

for (j=1;j<PeakCnt2;j++)

{

Peak2 = ExtractWord(j,PeakList2,";");

ReadPPMRange(Peak2,Lowcs2,Upcs2);

if( ((Lowcs2<=Lowcs1) && (Lowcs1<=Upcs2)) || ((Lowcs2<=Upcs1) && (Upcs1<=Upcs2)) || ((Lowcs1<=Lowcs2) && (Lowcs2<=Upcs1)) ) OvlpCnt++;

}

}

if (PeakCnt1 < PeakCnt2) OvlpLvl = OvlpCnt/(PeakCnt1-1.0);

else OvlpLvl = OvlpCnt/(PeakCnt2-1.0);

return OvlpLvl;

}

bool SwapMat(int ColNum1, int ColNum2, TStringList PeakList, TStringList SampleName, TStringList Page)

{

string chkstr;

chkstr = PeakList[ColNum1];

PeakList[ColNum1] = PeakList[ColNum2];

PeakList[ColNum2] = chkstr;

chkstr = SampleName[ColNum1];

SampleName[ColNum1] = SampleName[ColNum2];

SampleName[ColNum2] = chkstr;

chkstr = Page[ColNum1];

Page[ColNum1] = Page[ColNum2];

Page[ColNum2] = chkstr;

return true;

}

bool CalcOvlp(TStringList PeakList,TStringList SampleName,TStringList Page)

{

TStringList OMixSet,OPageSet;

int IMixSize,IMixSize0;

string OMixName,OMixFile,OPageFile;

double FOvlpLvl;

string ScriptPath,chkstr,chkstr2;

int i,j,k,m,n,count,SmpCnt,flag;

OMixSet = TStringList.Create();

OPageSet = TStringList.Create();

ScriptPath = ExtractFilePath(GetScriptFileName);

IMixSize = StrToInt(Lookup(ScriptPath+"Setting.txt","IMixSize"));

FOvlpLvl = StrToFloat(Lookup(ScriptPath+"Setting.txt","FOvlpLvl"));

SmpCnt = SampleName.Count();

OMixFile = Lookup(ScriptPath+"Setting.txt","OMixFile");

OMixName = Lookup(ScriptPath+"Setting.txt","OMixName");

OPageFile = Lookup(ScriptPath+"Setting.txt","OPageFile");

/* if samples i to j ovelap < FOvlpLvl, they will stay in the same mixture;

if sample j overlaps with any sample from i to j-1 > FOvlpLvl, we will search sample m

(m > j) till one overlaps with samples i to j-1 < FOvlpLvl, then swap sample j and m;

*/

count=1;

IMixSize0=IMixSize;

for (i=0;i<SmpCnt;i=i+IMixSize)

{

LogInfo("Mixture "+IntToStr(i));

IMixSize=IMixSize0;

for(j=i+1;(j<i+IMixSize)&&(j<SmpCnt);j++)

{

flag = 0;

for(k=i;k<j;k++) if(GetOvlpLvl(PeakList[j],PeakList[k]) > FOvlpLvl) {flag = 1;break;}

if (flag == 1)

{

for( m = j+1;m<SmpCnt;m++)

{

flag = 0;

for(k=i;k<j;k++) if(GetOvlpLvl(PeakList[m],PeakList[k])>FOvlpLvl) {flag = 1; break;}

if (flag == 0) break;

}

if(m<SmpCnt) SwapMat(j,m,PeakList,SampleName,Page);

}

if(m==SmpCnt) IMixSize=j-i;

}

chkstr = OMixName+IntToStr(count);

chkstr2 = chkstr;

for(k=i;(k<i+IMixSize)&&(k<SmpCnt);k++)

{

chkstr=chkstr+","+SampleName[k];

chkstr2 = chkstr2 + "," + Page[k];

}

OMixSet.Add(chkstr);

OPageSet.Add(chkstr2);

count++;

// print the overlap matrix;

LogInfo(chkstr);

for(k=i;(k<i+IMixSize+1)&&(k<SmpCnt);k++)

{

chkstr = "";

for(m=i;(m<i+IMixSize+1)&&(m<SmpCnt);m++) chkstr=chkstr+FormatFloat("0.00",GetOvlpLvl(Peaklist[k],PeakList[m]))+" ";

LogInfo(chkstr);

}

}

OMixSet.SaveToFile(OMixFile);

OPageSet.SaveToFile(OPageFile);

OMixSet.Free();

OPageSet.Free();

return true;

}

bool ReadPPMRange(string chkstr, double& Lowppm, double& Highppm)

{

int j;

chkstr=ExtractWord(2,chkstr,"[");

chkstr=ExtractWord(1,chkstr,"]");

j=Pos("..",chkstr);

Lowppm=StrToFloat(Trim(Copy(chkstr,1,j-1)));

Highppm=StrToFloat(Trim(Copy(chkstr,j+2,Length(chkstr)-j-1)));

return true;

}

bool ReadPeak(TSpecDocument Doc1D, TStringList PeakList)

{

TSpecmanTable TabInt;

double ILowCutOff,IHighCutOff;

string ScriptPath,chkstr;

double Lowppm,Highppm;

int i,rowcnt,IDppm;

ScriptPath = ExtractFilePath(GetScriptFileName);

TabInt = Doc1D.GetTableByName("Table of Integrals");

rowcnt = TabInt.RowsCount();

if (rowcnt < 1) {TabInt.Free(); return false;}

IDppm = TabInt.FindColumnIndex("(ppm)");

ILowCutOff = StrToFloat(Lookup(ScriptPath+"Setting.txt","ILowCutOff"));

IHighCutOff = StrToFloat(Lookup(ScriptPath+"Setting.txt","IHighCutOff"));

chkstr="";

for(i=0;i<rowcnt;i++)

{

ReadPPMRange(TabInt.GetValue(i,IDppm),Lowppm,Highppm);

if( (Lowppm>ILowCutOff) && (Highppm<IHighCutOff) ) chkstr = chkstr+TabInt.GetValue(i,IDppm)+";";

}

if (chkstr == "") {TabInt.Free(); return false;}

LogInfo(chkstr);

PeakList.Add(chkstr);

TabInt.Free();

return true;

}

String Lookup(String InitFileName, String ParName)

{

TStringList Setting;

int i,j,k,DimStr,DimDelim=4;

string chkstr,Delim[4];

Setting=TStringList.Create();

Setting.LoadFromFile(InitFileName);

DimStr=Setting.count;

Delim[0]=",";

Delim[1]=" ";

Delim[2]=chr(9);

Delim[3]=";";

for (i=0;i<DimStr;i++)

{

chkstr=Setting[i];

k=-1;

for(j=0;j<DimDelim;j++)

{

k = Pos(Delim[j],chkstr);

if (k>0) break;

}

if (k>0) if (Trim(Left(chkstr,k-1))==ParName) break;

}

Setting.Free();

return Trim(Right(chkstr,Length(chkstr)-k));

}

bool SortByIndex(TStringList Page, pointer& Index)

{

TStringList SortList;

int i,SmpCnt;

SmpCnt = Page.Count();

SortList = TStringList.Create();

for(i=0;i<SmpCnt;i++) SortList.Add(Page[Index[i]]);

for(i=0;i<SmpCnt;i++) Page[i]=SortList[i];

SortList.Free();

return true;

}

bool SortStrList(TStringList Page, pointer& Index)

{

int i,j,SmpCnt,tmp,flag;

string chkstr;

SmpCnt = Page.Count();

SetLength(Index,SmpCnt);

for (i=0;i<SmpCnt;i++) Index[i]=i;

for(i=0;i<SmpCnt;i++)

{

for(j=i+1;j<SmpCnt;j++)

{

flag = CompareText(Page[i],Page[j]);

if (flag>0)

{

chkstr=Page[i];

Page[i]=Page[j];

Page[j]=chkstr;

tmp=Index[i];

Index[i]=Index[j];

Index[j]=tmp;

}

}

LogInfo(Page[i]);

}

return true;

}

//main function

{

TSpecManagerDatabase LocalDB;

TSpecDocument Doc1D;

TStringList PeakList,SampleName,Page;

string FMonitorFID;

string ScriptPath;

int i,count,RecNum,SmpCnt,Index[255];

LocalDB = TspecManagerDatabase.OpenLocal(WorkFileName,false,"","");

if (LocalDB == nil)

{

LogInfo("Error: cannot load NMR database!");

exit;

}

PeakList = TStringList.Create();

SampleName = TStringList.Create();

Page = TStringList.Create();

ScriptPath = ExtractFilePath(GetScriptFileName);

FMonitorFID = Lookup(ScriptPath+"Setting.txt","FMonitorFID");

RecNum = LocalDB.RecordsCount();

count=0;

i=1;

while (i <= RecNum)

{

Doc1D = LocalDB.GetSpecDocument(i);

if (Doc1D == nil) break;

ExpName = Doc1D.GetUserData("Experimental Name","DOCUMENT");

if(FMonitorFID != ExpName) { i++; continue; }

if (ReadPeak(Doc1D,PeakList))

{

count++;

SampleName.Add(Doc1D.GetUserData("Sample Name","DOCUMENT"));

Page.Add(Doc1D.GetUserData("Project Name","DOCUMENT")+"_"+Doc1D.GetUserData("Page","DOCUMENT"));

}

i++;

}

SortStrList(SampleName,Index);

SortByIndex(Page,Index);

SortByIndex(PeakList,Index);

SampleName.SaveToFile(ScriptPath+"SortSampleName.txt");

Page.SaveToFile(ScriptPath+"SortPage.txt");

PeakList.SaveToFile(ScriptPath+"SortPeakList.txt");

/*

Page.LoadFromFile(ScriptPath+"SortPage.txt");

SampleName.LoadFromFile(ScriptPath+"SortSampleName.txt");

PeakList.LoadFromFile(ScriptPath+"SortPeakList.txt");

*/

CalcOvlp(PeakList,SampleName,Page);

CloseDocument(Doc1D);

PeakList.Free();

SampleName.Free();

Page.Free();

LocalDB.Free();

}

**Script S6.** Script for the processing and visualization of the fragment spectra of Watergate, STD and WaterLOGSY.

/*

ACD/Automation Script features:

1. Visualization and database for NMR fragment based screening;

2. One record generated for each mixture, including NMR experiment (Watergate, STD, WaterLogsy and CPMG) and the reference spectra;

3. File system independent. All settings will be loaded from Setting.txt file;

4. Read and process multiple FIDs automatically;

5. All input, output and judgement are controlled by parameters in Setting.txt

with the first letter of I, O and F, respectively;

Ke Ruan Copyright (c) 2012.

kruan@ustc.edu.cn

*/

String PlateName, MolName, EmailAddr, UserID, ExpName,FIDName; //reserved global variables.

int RecID;

/* update the primary screening data to the secondary confirmation for deconvolution check */

bool UploadPrimScr(TSpecManagerDatabase LocalDB)

{

TSpecManagerDatabase PrimDB;

TSpecDocument PrimDoc;

TIDList rec_ids, SpecMolIDList,ResultList;

string IPrimDBPath,ScriptPath,OMixName;

string chkstr;

int count,i;

ScriptPath=ExtractFilePath(GetScriptFileName);

OMixName = Lookup(ScriptPath+"Setting.txt","OMixName");

if(pos(OMixName,FIDName) != 0) return false;

chkstr = Lookup(ScriptPath+"Setting.txt","IPrimDBPath");

IPrimDBPath = chkstr + Lookup(ScriptPath+"Setting.txt",PlateName)+".nd9";

if(!FileExists(IPrimDBPath) && (pos("_",PlateName)!= 0) )IPrimDBPath = chkstr + ExtractWord(1,PlateName,"_")+".nd9";

if(!FileExists(IPrimDBPath)) return false;

rec_ids = TIDList.Create();

SpecMolIDList = TIDList.Create();

PrimDB=TSpecManagerDatabase.OpenLocal(IPrimDBPath,true,"","");

if (!PrimDB.SearchByUserData("'Any Field' Includes "+FIDName,rec_ids,dsb_Spectrum,SpecMolIDList))

{

rec_ids.Free();

SpecMolIDList.Free();

PrimDB.Free();

return false;

}

ResultList = TIDList.Create();

PrimDB.GetRecordSpecIDList(rec_ids[0],ResultList);

count = ResultList.Count;

for(i=0;i<count;i++)

{

PrimDoc = PrimDB.GetSpecDocument(ResultList[i]);

if(pos(OMixName,PrimDoc.GetUserData("Sample Name","DOCUMENT"))) LocalDB.UpdateSpecDocument2(PrimDoc,RecID);

}

rec_ids.Free();

SpecMolIDList.Free();

ResultList.Free();

PrimDoc.Free();

PrimDB.Free();

return true;

}

bool ProcessDoc(TSpecDocument Doc, string FIDPath,string DocNum, TChemicalStructure MolStructure)

{

TStringList MacCmd;

string IMacroName,MacroRes,ScriptPath,chkstr;

int np,ni,i,j;

if (Doc == nil) return false;

if(MolStructure != nil) Doc.AddChemicalStructure(MolStructure);

ScriptPath=ExtractFilePath(GetScriptFileName);

IMacroName = Lookup(ScriptPath+"Setting.txt","IMacroName"+DocNum);

IMacroName = ReplacePar(IMacroName);

if (Pos("\\",IMacroName) ==0 ) IMacroName=ScriptPath+IMacroName;

MacCmd=TStringList.Create();

chkstr = Doc.GetParameter("Original Points Count");

if (Pos("(",chkstr)==0) //1D NMR

{

np = StrToInt(chkstr);

np = np*2;

i=1024;

while (i<np) i=i*2;

Doc.ProcessMacroCommand("ZeroFilling (PointsCount = \""+IntToStr(i)+"\")",MacroRes);

}

/* else //2D NMR, to be optimized

{

chkstr=ExtractWord(2,chkstr,"(");

chkstr=ExtractWord(1,chkstr,")");

np=StrToInt(Trim(ExtractWord(1,chkstr,",")));

ni=StrToInt(Trim(ExtractWord(2,chkstr,",")));

i=32;

j=32;

while(i<2*np) i=i*2;

while(j<4*ni) j=j*2;

Doc.ProcessMacroCommand("FullFT (Dataset2 = \"Complex\"; Apodization2 = \"User\"; EM2 = \"20.00, 0.000\"; GM2 = \"Off\"; Sine2 = \"Off\"; SqSine2 = \"Off\"; InitialSize2 = "+IntToStr(np)+"; FinalSize2 = "+IntToStr(i)+"; Backward2 = \"off\"; Forward2 = \"Off\"; FIDShift2 = \"Default\"; FirstPointScalingFactor2 = 0.50; Dataset1 = \"1, 0, 1, 0, 0, -1, 0, 1\"; Apodization1 = \"User\"; EM1 = \"20.00, 0.000\"; GM1 = \"Off\"; Sine1 = \"Off\"; SqSine1 = \"Off\"; InitialSize1 = "+IntToStr(ni)+"; FinalSize1 = "+IntToStr(2*j)+"; Backward1 = \"Off\"; Forward1 = \""+IntToStr(ni+1)+", "+IntToStr(j)+", 1, "+IntToStr(ni)+", 8\"; FIDShift1 = \"Default\"; FirstPointScalingFactor1 = 0.50)",MacroRes);

}

*/

if(FileExists(IMacroName))

{

MacCmd.LoadFromFile(IMacroName);

Doc.ProcessMacroCommandsList(MacCmd,MacroRes);

}

MacCmd.Free();

return true;

}

//Copy raw data to a user defined directory to avoid ACD scanning over those files repeatedly.

bool CopyNMR(string FIDPath)

{

string OFIDPath,ScriptPath,chkstr,FileExt;

int i;

ScriptPath = ExtractFilePath(GetScriptFileName);

OFIDPath = Lookup(ScriptPath+"Setting.txt","OFIDPath");

OFIDPath = ReplacePar(OFIDPath);

i=1;

if (OFIDPath == "") return false;

if (!DirExists(OFIDPath))

{

CreateDir(OFIDPath,true);

RmDir(OFIDPath);

}

else

{

chkstr=OFIDPath;

FileExt = ExtractFileExt(OFIDPath);

while (DirExists(chkstr))

{

chkstr = StringReplace(OFIDPath,FileExt,"_0"+IntToStr(i)+FileExt);

i++;

}

OFIDPath=chkstr;

}

CopyDir(FIDPath,OFIDPath);

if(FileExists(OFIDPath+"\\fid")) RmDir(FIDPath);

return true;

}

bool SetPar(TSpecDocument Doc,String FIDPath)

{

string chkstr,MacroRes;

chkstr = ReadPar(FIDPath+"procpar","page");

Doc.ProcessMacroCommand("SetUserData (Name = \"Page\"; Value = \""+chkstr+"\"; Type = \"Spectrum\")",MacroRes);

Doc.ProcessMacroCommand("SetUserData (Name = \"User ID\"; Value = \""+UserID+"\"; Type = \"Spectrum\")",MacroRes);

Doc.ProcessMacroCommand("SetUserData (Name = \"Email Address\"; Value = \""+EmailAddr+"\"; Type = \"Spectrum\")",MacroRes);

Doc.ProcessMacroCommand("SetUserData (Name = \"Project Name\"; Value = \""+PlateName+"\"; Type = \"Spectrum\")",MacroRes);

Doc.ProcessMacroCommand("SetUserData (Name = \"Sample Name\"; Value = \""+FIDName+"\"; Type = \"Spectrum\")",MacroRes);

Doc.ProcessMacroCommand("SetUserData (Name = \"Structure Name\"; Value = \""+MolName+"\"; Type = \"Spectrum\")",MacroRes);

return true;

}

string ReplacePar(string ParName)

{

string chkstr;

chkstr = StringReplace(ParName,"ParPlateName",PlateName);

chkstr = StringReplace(chkstr,"ParMolName",MolName);

chkstr = StringReplace(chkstr,"ParEmailAddr",EmailAddr);

chkstr = StringReplace(chkstr,"ParUserID",UserID);

chkstr = StringReplace(chkstr,"ParExpName",ExpName);

chkstr = StringReplace(chkstr,"ParFIDName",FIDName);

return chkstr;

}

string ReadPar(string ProcparName, string ParName)

{

TStringList ParFile;

int i,j,count;

string chkstr;

ParFile=TStringList.Create();

if(!ParFile.LoadFromFile(ProcparName))

{

LogInfo("Error: can not open procpar file.");

return "";

}

i=0;

count=ParFile.Count();

while (i<count)

{

chkstr=ParFile[i];

j=Pos(" ",chkstr);

if (Trim(Left(chkstr,j))==ParName)

{

ParName = ExtractWord(2,ParFile[i+1],"\"");

if (ParName == "") ParName = ExtractWord(2,ParFile[i+1]," ");

break;

}

i++;

}

ParFile.Free();

return ParName;

}

String Lookup(String InitFileName, String ParName)

{

TStringList Setting;

int i,j,k,DimStr,DimDelim=4,flag;

string chkstr,Delim[4];

Setting=TStringList.Create();

Setting.LoadFromUnicodeFile(InitFileName);

DimStr=Setting.count;

flag=0;

k=Length(ParName);

for (i=0;i<DimStr;i++)

{

chkstr=Setting[i];

if(Left(chkstr,k)==ParName) {flag=1; break;}

}

Setting.Free();

if (flag==1) return Trim(Right(chkstr,Length(chkstr)-k));

else return "";

}

//Replace wildcard in the filename

string ReplaceWildCard(string FileName)

{

TStringList NameList;

string tmpname,chkstr;

int i,count;

if (Pos("*",FileName)==0) return FileName;

NameList = TStringList.Create();

count = WordCount(FileName,"\\");

chkstr = "";

for(i=1;i<count;i++)

{

tmpname = ExtractWord(i,FileName,"\\");

if (Pos("*",tmpname)==0) chkstr = chkstr+tmpname+"\\";

else

{

ExecApp2("CMD","/C DIR /B "+chkstr+tmpname,NameList);

if(NameList.Count() ==1) chkstr = chkstr + NameList[0]+"\\";

else

{

NameList.Free();

LogInfo("FileName Incorrect!");

return "";

}

}

}

tmpname = ExtractWord(count,FileName,"\\");

if (Pos("*",tmpname)==0) FileName = chkstr+tmpname;

else

{

ExecApp2("CMD","/C DIR /B "+chkstr+tmpname,NameList);

if(NameList.Count() ==1) FileName = chkstr + NameList[0];

else

{

NameList.Free();

LogInfo("FileName Incorrect!");

return "";

}

}

NameList.Free();

return FileName;

}

bool InitSetting(String FIDPath)

{

string ScriptPath;

//Load 5 global variables;

PlateName = "";

EmailAddr = "";

UserID = "";

MolName = "";

FIDName = "";

ExpName = "";

ScriptPath=ExtractFilePath(GetScriptFileName);

PlateName = Lookup(ScriptPath+"Setting.txt","ParPlateName");

EmailAddr = Lookup(ScriptPath+"Setting.txt","ParEmailAddr");

UserID = Lookup(ScriptPath+"Setting.txt","ParUserID");

MolName = Lookup(ScriptPath+"Setting.txt","ParMolName");

FIDName = Lookup(ScriptPath+"Setting.txt","ParFIDName");

ExpName = Lookup(ScriptPath+"Setting.txt","ParExpName");

//enumerate the global variable from procpar

if (!FileExists(FIDPath+"procpar"))

{

LogInfo("Error: procpar file not found.");

return false;

}

if(PlateName!="") PlateName = ReadPar(FIDPath+"procpar",PlateName);

if(UserID!="") UserID = ReadPar(FIDPath+"procpar",UserID);

if(MolName!="") MolName = ReadPar(FIDPath+"procpar",MolName);

if(FIDName!="") FIDName = ReadPar(FIDPath+"procpar",FIDName);

if(ExpName!="") ExpName = ReadPar(FIDPath+"procpar",ExpName);

if(EmailAddr!="")

{

EmailAddr = ReadPar(FIDPath+"procpar",EmailAddr);

EmailAddr = StringReplace(EmailAddr,"_",".");

}

return true;

}

/************* Main script *********************/

{

TSpecDocument Doc1,Doc2,Doc3,Doc,Doc4;

TSpecManagerDatabase LocalDB;

TChemicalStructure MolStructure;

TSDFile SDFStructure;

string FMonitorFID,ODBPath;

string IMacroName,IFIDName,IMixFile,IRefMode, IMolPath, ISDFPath,ISDFCompoundName;

string FIDPath,ScriptPath,chkstr,MacroRes,RefName,Delim[4];

int i,j,count,DimDelim=4,SDIndex;

FIDPath=ExtractFilePath(WorkFileName);

// LogInfo(FIDPath);

if(!InitSetting(FIDPath))

{

LogInfo("Initialization Failed!");

exit;

}

ScriptPath=ExtractFilePath(GetScriptFileName);

FMonitorFID = Lookup(ScriptPath+"Setting.txt","FMonitorFID");

FMonitorFID = ReplacePar(FMonitorFID);

if (pos(FMonitorFID,ExpName)==0)

{

LogInfo("Error: Inappropriate 1D File Name!");

exit;

}

MolStructure=TChemicalStructure.Create();

IMolPath = Lookup(ScriptPath+"Setting.txt","IMolPath");

IMolPath = ReplacePar(IMolPath);

if (Pos("\\",IMolPath) == 0) IMolPath = FIDPath+IMolPath;

IMolPath=StringReplace(IMolPath,"PXP_","");

IMolPath = ReplaceWildCard(IMolPath);

if(FileExists(IMolPath)) MolStructure.LoadFromMolFile(IMolPath);

else

{

ISDFPath = Lookup(ScriptPath+"Setting.txt","ISDFPath");

ISDFPath = ReplacePar(ISDFPath);

ISDFPath = ReplaceWildCard(ISDFPath);

if (FileExists(ISDFPath))

{

SDFStructure = TSDFile.Open(ISDFPath,false);

ISDFCompoundName = Lookup(ScriptPath+"Setting.txt","ISDFCompoundName");

SDFStructure.FindIndexByData(ISDFCompoundName,MolName,SDIndex,false,0);

if (SDIndex >0) MolStructure = SDFStructure.ReadStructure(SDIndex);

}

if (MolStructure.AtomsCount()<1)

{

ISDFPath = Lookup(ScriptPath+"Setting.txt","IGlobalSDF");

ISDFPath = ReplacePar(ISDFPath);

ISDFPath = ReplaceWildCard(ISDFPath);

if (FileExists(ISDFPath))

{

SDFStructure = TSDFile.Open(ISDFPath,false);

ISDFCompoundName = Lookup(ScriptPath+"Setting.txt","ISDFCompoundName");

SDFStructure.FindIndexByData(ISDFCompoundName,MolName,SDIndex,false,0);

if (SDIndex >0) MolStructure = SDFStructure.ReadStructure(SDIndex);

}

}

}

Doc1=ImportDocument(WorkFileName);

FIDPath = ExtractFilePath(WorkFileName);

SetPar(Doc1,FIDPath);

ProcessDoc(Doc1,FIDPath,"1",MolStructure);

CopyNMR(FIDPath);

/* Process cpmg spectrum */

IFIDName = Lookup(ScriptPath+"Setting.txt","IFIDName2");

IFIDName = Replacepar(IFIDName);

IFIDName = ReplaceWildCard(IFIDName);

if ( FileExists(IFIDName) )

{

FIDPath = ExtractFilePath(IFIDName);

if(!InitSetting(FIDPath))

{

LogInfo("Initialization Failed!");

exit;

}

Doc2=ImportDocument(IFIDName);

SetPar(Doc2,FIDPath);

ProcessDoc(Doc2,FIDPath,"2",MolStructure);

CopyNMR(FIDPath);

}

/* Process std spectrum */

IFIDName = Lookup(ScriptPath+"Setting.txt","IFIDName3");

IFIDName = Replacepar(IFIDName);

IFIDName = ReplaceWildCard(IFIDName);

if ( FileExists(IFIDName) )

{

FIDPath = ExtractFilePath(IFIDName);

if(!InitSetting(FIDPath))

{

LogInfo("Initialization Failed!");

exit;

}

Doc3=ImportDocument(IFIDName);

SetPar(Doc3,FIDPath);

ProcessDoc(Doc3,FIDPath,"3",MolStructure);

CopyNMR(FIDPath);

}

/* Process waterlogsy spectrum */

IFIDName = Lookup(ScriptPath+"Setting.txt","IFIDName4");

IFIDName = Replacepar(IFIDName);

IFIDName = ReplaceWildCard(IFIDName);

if ( FileExists(IFIDName) )

{

FIDPath = ExtractFilePath(IFIDName);

if(!InitSetting(FIDPath))

{

LogInfo("Initialization Failed!");

exit;

}

Doc4=ImportDocument(IFIDName);

SetPar(Doc4,FIDPath);

ProcessDoc(Doc4,FIDPath,"4",MolStructure);

CopyNMR(FIDPath);

}

RecID=-1;

ODBPath=Lookup(ScriptPath+"Setting.txt","ODBPath");

ODBPath=ReplacePar(ODBPath);

LocalDB=TSpecManagerDatabase.OpenLocal(ODBPath,true,"","");

if (Doc3 != nil) LocalDB.UpdateSpecDocument2(Doc3,RecID);

LocalDB.UpdateSpecDocument2(Doc1,RecID);

if (Doc2 != nil) LocalDB.UpdateSpecDocument2(Doc2,RecID);

if (Doc4 != nil) LocalDB.UpdateSpecDocument2(Doc4,RecID);

//Process reference spectra

IMixFile = Lookup(ScriptPath+"Setting.txt","IMixFile");

IMixFile = Replacepar(IMixFile);

IRefMode = Lookup(ScriptPath+"Setting.txt","IRefMode");

IRefMode = Replacepar(IRefMode);

chkstr = Lookup(IMixFile,FIDName);

LogInfo(FIDName);

LogInfo(chkstr);

count=0;

Delim[0]=",";

Delim[1]=" ";

Delim[2]=chr(9);

Delim[3]=";";

for (i=0;i<DimDelim;i++)

{

count = WordCount(chkstr,Delim[i]);

if (count>1) break;

}

for(j=0;j<count;j++)

{

RefName = ExtractWord(j+1,chkstr,Delim[i]);

LogInfo(RefName);

RefName = StringReplace(IRefMode,"ParRefName",RefName);

if(FileExists(RefName))

{

Doc = ImportDocument(RefName);

FIDPath = ExtractFilePath(RefName);

IMacroName = Lookup(ScriptPath+"Setting.txt","IMacroNameRef");

if(IMacroName != "") ProcessDoc(Doc,FIDPath,"Ref",MolStructure);

Doc.ProcessMacroCommand("SetUserData (Name = \"Hit\"; Value = \"n\"; Type = \"Spectrum\")",MacroRes);

LocalDB.UpdateSpecDocument2(Doc,RecID);

}

}

UploadPrimScr(LocalDB);

CloseDocument(Doc1);

if (Doc2!= nil) CloseDocument(Doc2);

if (Doc3 != nil) CloseDocument(Doc3);

if (Doc4 != nil) CloseDocument(Doc4);

LocalDB.Free();

}
